# Phylogenetic Comparison and Splicing Analysis of the U1 snRNP-specific Protein U1C in Eukaryotes

**DOI:** 10.3389/fmolb.2021.696319

**Published:** 2021-09-09

**Authors:** Kai-Lu Zhang, Jian-Li Zhou, Jing-Fang Yang, Yu-Zhen Zhao, Debatosh Das, Ge-Fei Hao, Caie Wu, Jianhua Zhang, Fu-Yuan Zhu, Mo-Xian Chen, Shao-Ming Zhou

**Affiliations:** ^1^ Division of Gastroenterology, Shenzhen Children’s Hospital, Shenzhen, China; ^2^ Co-Innovation Center for Sustainable Forestry in Southern China, College of Biology and the Environment, Nanjing Forestry University, Nanjing, China; ^3^ State Key Laboratory of Crop Biology, College of Life Science, Shandong Agricultural University, Taian, China; ^4^ Key Laboratory of Pesticide and Chemical Biology, Ministry of Education, College of Chemistry, Central China Normal University, Wuhan, China; ^5^ College of Light Industry and Food Engineering, Nanjing Forestry University, Jiangsu, China; ^6^ Department of Biology, Hong Kong Baptist University, and State Key Laboratory of Agrobiotechnology, The Chinese University of Hong Kong, Hong Kong, SAR China

**Keywords:** alternative splicing, phylogenetics, gene expression, splice site, U1-snRNP

## Abstract

As a pivotal regulator of 5’ splice site recognition, U1 small nuclear ribonucleoprotein (U1 snRNP)-specific protein C (U1C) regulates pre-mRNA splicing by interacting with other components of the U1 snRNP complex. Previous studies have shown that U1 snRNP and its components are linked to a variety of diseases, including cancer. However, the phylogenetic relationships and expression profiles of U1C have not been studied systematically. To this end, we identified a total of 110 animal *U1C* genes and compared them to homologues from yeast and plants. Bioinformatics analysis shows that the structure and function of U1C proteins is relatively conserved and is found in multiple copies in a few members of the U1C gene family. Furthermore, the expression patterns reveal that U1Cs have potential roles in cancer progression and human development. In summary, our study presents a comprehensive overview of the animal U1C gene family, which can provide fundamental data and potential cues for further research in deciphering the molecular function of this splicing regulator.

## Introduction

Discovered in the late 1970s, precursor messenger RNA (pre-mRNA) splicing, including constitutive splicing and alternative splicing, is an important biological process in eukaryotes (van den Hoogenhof et al., 2016) (Berget et al., 1977). This process is performed by a large protein complex called the spliceosome, which removes noncoding sequences (introns) and ligates functional coding sequences (exons) to generate mature mRNAs (Ellis et al., 2008). Spliceosomes are composed of multiple proteins and several Uridine(U)-rich small nuclear ribonucleoproteins (snRNPs), including U1, U2, U4/U6, U5, U11 and U12 (Chen and Moore, 2015). Splicing machinery is assembled in a stepwise manner by different spliceosomal snRNPs (Wahl et al., 2009). The U1 snRNP-mediated recognition of downstream 5’ splice sites of introns is the first step accomplished by a subcomplex during spliceosome assembly: the subcomplex is composed of one U1 snRNA, 8-9 Sm proteins and three specific proteins, including U1-70K, U1A and U1C, in humans and yeast (Wahl et al., 2009) (Lehmeier et al., 1990). U1-70K and U1A can bind directly to U1 snRNA, while U1C alone cannot attach to U1 snRNA, and the binding of the U1 snRNP core domain and U1C needs to be mediated by U1-70K and Sm proteins (Du and Rosbash, 2002) (Nelissen et al., 1994). Importantly, the U1C protein is specifically associated with the Ul snRNP and plays a unique role in recognizing the 5’ splice site independently of base pairing (Du and Rosbash, 2002) (Heinrichs et al., 1990).

The biological functions of U1 snRNP have been characterized and shown to affect human pathogenesis and autoimmune diseases in addition to the 5’ SS recognition role. For instance, pathological examination, proteomics and transcriptomics have demonstrated that U1 snRNP components aggregate in neuronal cell bodies, resulting in a global disruption of RNA processing in Alzheimer’s disease (AD) brains (Bai et al., 2013). Furthermore, U1 snRNP is demonstrated to be linked to a molecular pathway associated with amyotrophic lateral sclerosis (ALS), indicating that splicing defects may play a key role in the pathogenesis of motor neuron disease, providing a potential therapeutic target (Yu et al., 2015). In addition, U1 snRNP is also reported to be associated with other diseases, such as autoimmunity connective tissue disease (MCTD), systemic lupus erythematosus, congenital myasthenic syndrome (CMS) and others (Chi et al., 2018) (Hof et al., 2005). All of these studies demonstrate that U1 snRNPs play important roles in human disease. As a vital component of the U1 snRNP, the phylogeny and splicing pattern of U1C remain unclear. Thus, in this study, we identified and analysed the phylogenetic relationships of the *U1C* gene family in different animal species. Genome-wide bioinformatics analysis, including elucidation of gene structures, protein domains, expression profiles and conserved splicing patterns, has been performed to explore the potential functions of *U1Cs*, providing theoretical support for further functional investigation. (Rahman et al., 2015)

### Experimental Methods

#### Sequence Identification and Collection of U1C Proteins in Animals

The U1C protein sequence (ENSP00000363129.3) (Satoh et al., 2011) from *Homo sapiens* was used as a query sequence to perform protein BLAST (e-value cut-off = 1e^−10^) to find similar sequences in all the available animal and yeast genomes (present in Ensembl genome browser 96 (http://asia.ensembl.org/index.html)) (Cunningham et al., 2019). The resulting sequences were screened for the PF06220 (U1 zinc finger, zf-U1) domain using the HMMSCAN algorithm implemented in HMMER 3.2.1 (Johnson et al., 2010), after which 110 protein sequences were retained.

### Construction of Phylogenetic Tree of *U1C* Genes

The above 110 proteins were used for multiple sequence alignment in Muscle v3.8 (Edgar, 2004) and for construction of a phylogenetic tree of animal *U1C* genes using PhyML v3.037 based on the maximum likelihood method with the JTT + G + F model (Guindon et al., 2010). Meanwhile, the sequences of plant *U1C* genes derived from our previous work (Yang et al., 2021 unpublished) were combined to build a tree of plants, yeast, and animals with FastTree (Price et al., 2010). The resulting phylogenetic trees were visualized in FigTree v1.4.3.38 (Vlad I et al., 2008).

### Analysis of Gene Structures, Protein Domains and MEME Motifs

Genomic, cDNA, CDS and peptide sequences for the above identified U1Cs were downloaded from the Ensembl database. Gene structure was reconstructed on Gene Structure Display Server 2.0 (GSDS2.0) (http://gsds.gao-lab.org/) (Hu et al., 2015). Protein domains were found using the HMMER website (https://www.ebi.ac.uk/Tools/hmmer/) (Potter et al., 2018) and were drawn using TBtools (Chen et al., 2020a). cDNA and protein sequences were used as inputs to predict the 10 most conserved motifs on the MEME (multiple Em for motif elicitation) server operated with the default parameters (http://meme-suite.org/tools/meme) (Bailey et al., 2009).

### Analysis of Protein Interaction Networks

Protein sequences of humans (ENSP00000363129.3), *Mus musculus* (ENSMUSP00000156644.1) and *Saccharomyces cerevisiae* (YLR298C_mRNA) were used as input to obtain the protein-protein interaction networks on the STRING web server (https://string-db.org/) (Szklarczyk et al., 2015) using its active interaction sources: experiments and databases. Finally, predicted functional partners (confidence cutoff of 0.900) of U1C proteins were presented in the form of an interaction network drawn by Cytoscape 3.8 software.

### Amino Acid Conservation Estimation

The crystal structure of human U1C protein (PDB ID: 4PJO) downloaded from the PDB database (Berman et al., 2000) was used as an input file in the ConSurf server to represent the evolutionary conservation of animal *U1C* genes. The corresponding model of plants was retrieved from a previous work. The residue numbers of human U1C are labelled according to the human sequence (ENSP00000363129.3) in the representative figure. Sequences with large gaps were deleted in this amino-acid (aa) conservation estimation. U1C from model plant *Arabidopsis* (At4g03120) was used for comparison against human U1C.

### AS Profile Analysis and Identification of Conserved Splice Sites

Sequences of all available splice isoforms of animal *U1C* genes were downloaded from the Ensembl database. Selected splice junction sequences (20 bp on each side) were aligned using the online webtool MultAlin (http://multalin.toulouse.inra.fr/multalin/multalin.html), and sequence logos were generated by Weblogo v2.8.2 (https://weblogo.berkeley.edu/logo.cgi) (Chen et al., 2020b) (Zhu et al., 2017).

### Expression Analysis From Online Microarray Datasets

Expression data based on baseline expression of human (Ensg00000124562) and mouse (Ensmusg00000024217) *U1C*s were downloaded from the Expression Atlas database (https://www.ebi.ac.uk/gxa/home). The heatmaps of retrieved expression data were generated by using the online BAR HeatMapper Tool (http://bar.utoronto.ca/ntools/cgi-bin/ntools_heatmapper.cgi) (Chen et al., 2020a).

## Results

### Phylogenetic Analysis of Animal *U1C* Genes

In this study, a total of 110 U1C protein sequences from 61 animal species, including 93 placentals (51 primates, 28 rodents and lagomorphs and 14 other mammals), 5 marsupials, monotremes and reptiles, one other vertebrate (*Xenopus tropicalis*), 7 fish, and 4 other species, were identified (Table S1). More than half of the members of the U1C family involved multiple gene copies (72/110), including twelve species with two copies, seven species with three copies and five species with four copies. In particular, seven copies were found in Ma’s night monkey (*Aotus nancymaae*). Only 38 animal species contained one copy of *U1C*.

**FIGURE 1:**
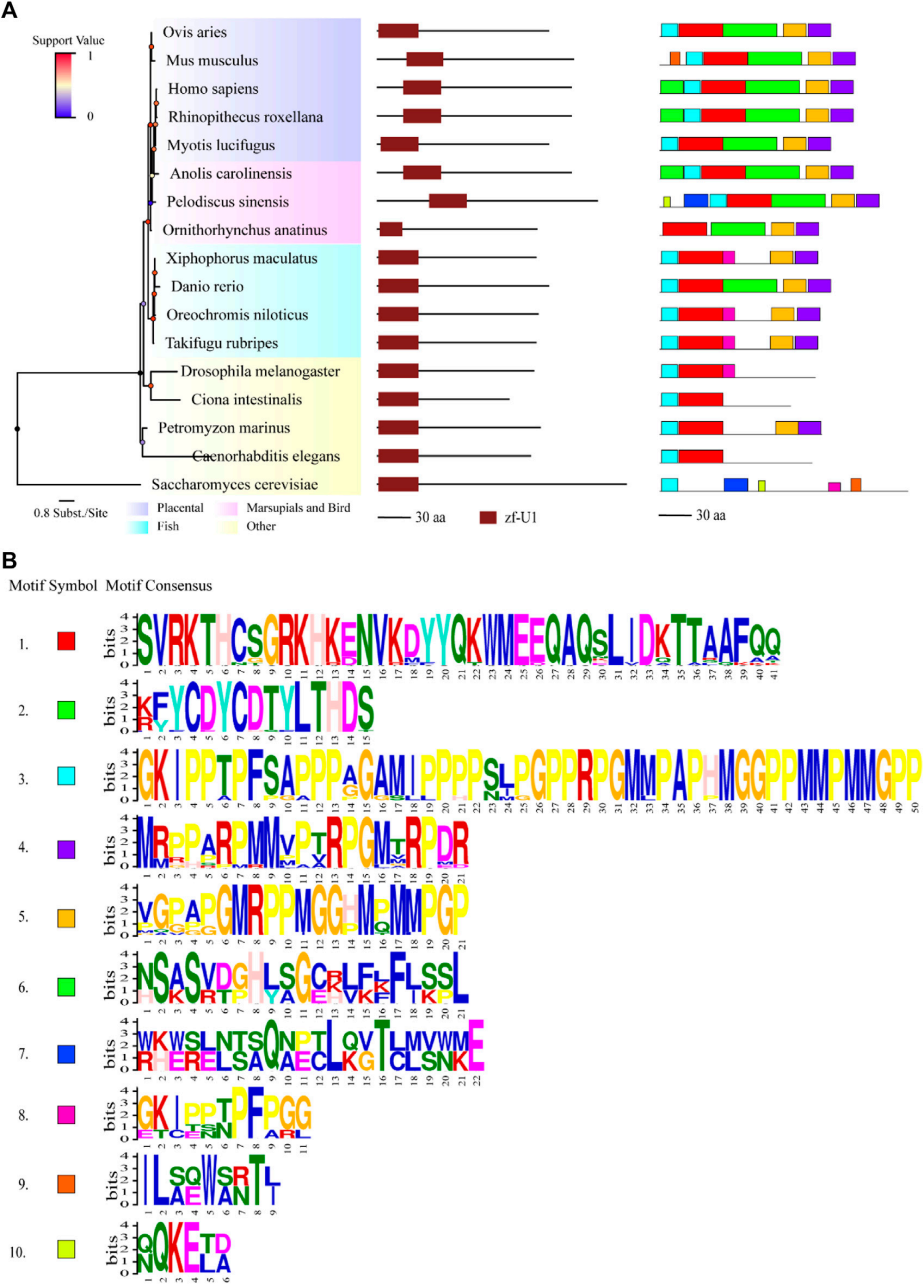
Comparisons of structure and motif analysis of animal representative U1C proteins. **(A)** The vertical phylogenetic tree based on representative 17 U1C proteins is listed on the left panel. The legend on the top left corner represents support value of the tree. Blue/0 to red/1 means the range of values. The length of the scale bar corresponds to 0.8 substitutions per site. The predicted protein domains (zf-U1) by online HMMER are listed on the middle panel. The identified protein conserved motifs with MEME analysis are shown on the right panel. The 10 most frequently discovered motifs are shown in different solid colors and additional scanned sites (*p*-values less than 0.0001) are shown in transparent color. **(B)** The logos and detailed information of ten motifs from MEME are shown below. The height of symbols indicates the degree frequency of each amino acid at that position. U1C sequences from human, mouse and *C. elegens* and yeast are highlighted by red arrow.

To understand the evolutionary history and phylogenetic relationships between the above 110 identified *U1C* genes, a rooted phylogenetic tree of the family was constructed based on multiple protein sequences alignment (Supplementary Figure S4, left panel and Supplementary Figure S2), showing four major clades, namely, placentals (purple), marsupials, monotremes and reptiles (pink), fish (light blue) and other species (yellow). Furthermore, five members of the yellow clade (other species) with longer branch length formed the basal part of the circle phylogenetic tree, suggesting its distant association with other clades. More specifically, Xenopus (*X. tropicalis*, ENSXETP00000053216.1) gathers to one branch point with other vertebrates (placentals, marsupials, monotremes, reptiles and Xenopus), which suggests that lamprey is a significant link that connects vertebrates and invertebrates (Supplementary Figure S4, left panel). Moreover, placentals, marsupials, monotremes and reptiles formed a sister clade with Xenopus (*X. tropicalis*, ENSXETP00000053216.1). In summary, the clustered four major clades reflected the general animal phylogeny (Supplementary Figure S2 and Figure 1, left panel). Moreover, the length of a branch indicates the evolutionary distance between organisms and the clear topology suggested the validity of this phylogenetic reconstruction of the animal U1C gene family. The highly accurate phylogenetic tree constructed here may serve fundamentals for subsequent bioinformatic analysis.

### Protein Domain and Conserved Motifs Analysis

Protein domains and conserved motifs were analysed to further infer the functional properties of U1C proteins. Most of the U1Cs contained a domain called zf-U1 (PF06220), as predicted by the HMMER website*,* in addition to *Pan troglodytes* (ENSPTRP00000071198.1), *Macaca nemestrina* (ENSMNEP00000006688.1) and *Jaculus jaculus* (ENSJJAP00000018575.1), without any signatures in their sequences (Supplementary Figure S4, middle panel). The identified U1C proteins from all species were characterized to range from 99 to 231 amino acids in length (average length 163.2 aa) (Supplementary Table S2). To identify the specific conserved motifs in animal U1C proteins, the 10 most conserved motifs were predicted by using the MEME suite based on acquired animal U1C protein sequences (Figure 1 and Supplementary Figure S4, right panel). The results show that only 16 sequences of U1Cs, including human U1C, have the ten conserved motifs identified in this work, indicating the divergence of animal U1C proteins in terms of conserved domain content. Barring one sequence, ENSSHAP00000001057.1 (*Sarcophilus harrisii*) from marsupials, the other 15 sequences were from placentals. The zf-U1 domain was present in the first three motifs at the N-terminus of most U1Cs (Figure 1, right panel). Furthermore, most U1Cs from placentals contain nine conserved motifs, while those from marsupials, monotremes and reptiles have approximately eight or nine motifs, and the U1Cs of fish have approximately seven or eight motifs (Supplementary Figure S4, right panel). As expected, protein sequences from other species in the basal part of the phylogenetic tree had only one to five motifs and showed the least degree of conservation. In general, motifs 2, 6 and half of motif 1 together formed the region of the conserved zf-U1 domain, implying that these three motifs maybe evolved together along with the animal lineage. Furthermore, the majority of the U1C proteins contained motifs 4, 5 and 8 at C-terminus, suggesting that these three motifs may have additional function among these species.

### Conservation Analysis and Interaction Networks of U1C

**FIGURE 2:**
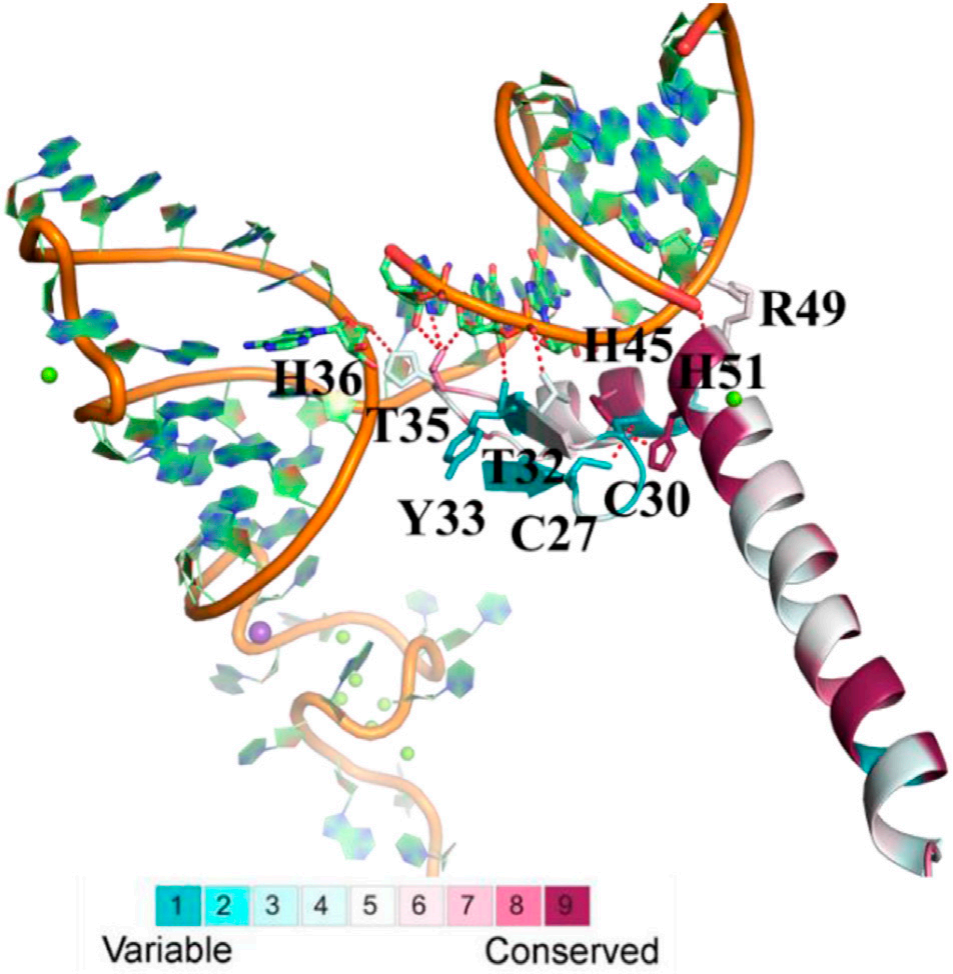
The evolutionary conservation analysis of amino acid positions in animal U1Cs. The crystal structure of human U1C with its target RNA is shown. The ribbon representation is colored according to ConSurf Grade (1-blue to 9-purple) by using all identified protein sequences of animal U1Cs. C27, C30, H36, H45, H51, R49, T32, T35 and Y33 are residues of human U1C. C, H, R, T and Y are symbol of cysteine, histidine, arginine, threonine and tyrosine, respectively.

Since the crystal structure of the human U1C (PDB ID: 4PJO, chain L) RRM domain is publicly available, domain evolutionary conservation analysis was based on this structure (the residue number of human U1C is shown in Figure 2 and Supplementary Figure S5). The ConSurf grade of 20 (39.2%) residues was over 7, and the ConSurf grade of 10 (19.6%) residues was over 9. Meanwhile, the conservation of 50 amino acids exceeded 90% among 51 sites, indicating the high conservation of this gene in animals.

In detail, His45 and His51 in the zinc-binding pocket are highly conserved, but Cys27 and Cys30 are not. The corresponding residues of Cys27 and Cys30 in ENSMFAP00000017249.1 (Phe17 and Phe20), ENSCANP00000009254.1 (Leu15 and Phe18), ENSANAP00000004977.1 (Cys18 and TYR21), and ENSSBOP00000033444.1 (Cys13 and Arg16) are partially or completely replaced by other residues, which may result in weak binding to the metal ion. On the other hand, the side chains of Thr32, Thr35, and His36 and the backbones of Tyr33 and Arg49 form hydrogen bonds with RNA. The mutation of Tyr33 and Arg49 may not reduce the binding affinity, but the change of residues at the positions of Thr32, Thr35, and His36 may influence it, as observed in *M. fascicularis* (ENSMFAP00000017249.1), *C. angolensis palliates* (ENSCANP00000009254.1), *Microcebus murinus* (ENSMICP00000032926.1), and *Otolemur garnettii* (ENSOGAP00000022219.1) (Li et al., 2017) (Muto et al., 2004). These genes all have paralogous genes in certain species with a conserved binding domain similar to other *U1C* genes. All of these results reveal that animal *U1C* genes are conserved except for some specific genes and that the biological function of these “specific genes” is redundant with other genes of the same organism.

Previous studies (Yang et al., 2021 unpublished) have suggested that plant *U1C* genes are conserved (shown in Supplementary Table S5). The conservation of animal and plant U1C was further compared in this study, and the multiple sequence alignment of animal and plant U1C sequences is shown in Supplementary Figure S3. It seems that the C-terminal residues of plants are less conserved than those of animals. For example, the residues at the positions of Asp57 and Glu64 in humans are less conserved (Supplementary Figure S5). Moreover, some residues are species-specific, such as Thr44/Q23, Cys46/Asn25, Ser47/Ala26, Arg49/Tyr28, Glu53/Ala32, Lys56/Arg35, Lys61/Gln40, Trp62/Phe41, Met63/Glu42, Glu65/Gln44, Ala67/Thr46, Lys73/Gln52, Thr74/Arg53, and Thr75/Ile54 are in animals/plants (residue number of *Arabidopsis* U1C). Only Thr44/Q23 is on the interface of the interaction surface between U1C and RNA. Q23 of *Arabidopsis* U1C prefers to interact with RNA with an extra hydrogen bond, which may improve the binding capability. The above predicted outcomes need to be further demonstrated by wet-lab experiments in the future. In conclusion, the 3D consensus model of animal U1C proteins demonstrated the conservation of this gene family in animals at the aspect of 3D-structure. Furthermore, divergence of particular amino acid may contribute to the functional divergence of these U1Cs in snRNA binding and recognition, which is worth to be studied in future investigation.

**FIGURE 3:**
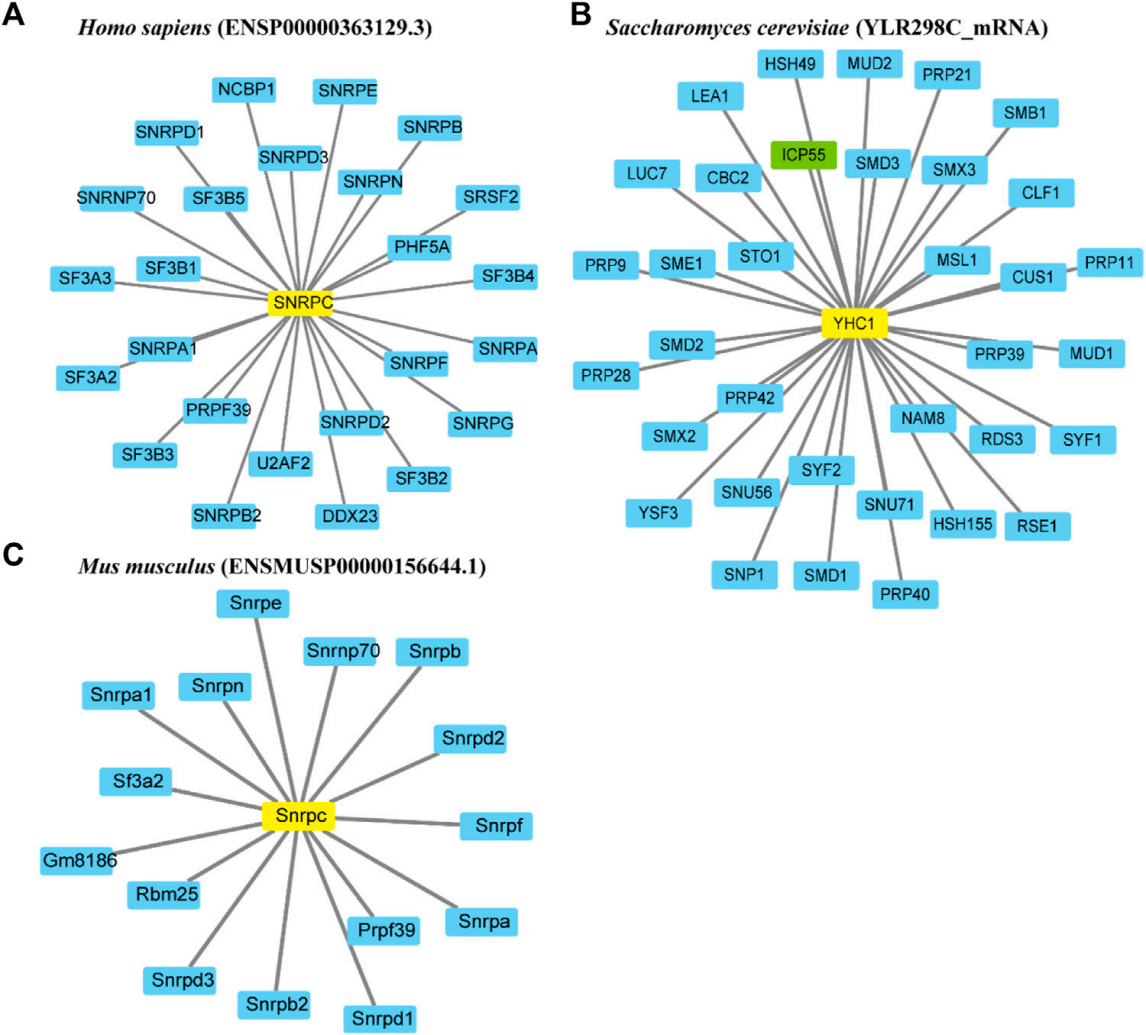
Representative protein-protein interaction networks of *Homo sapiens*, *Mus musculus* and *Saccharomyces cerevisiae*. Interaction networks of U1Cs (highlighted in yellow) of **(A)** human (ENSP00000363129.3), **(B)** mouse (ENSMUSP00000156644.1) and **(C)** yeast (YLR298C_mRNA) are carried out by Cytoscape 3.8 software based on experiments and databases from STRING database. Interactors involved in splicing are highlighted in blue, while ICP55 as the only gene not involved in splicing is shown in green. Minimum required interaction score (combined score): 0.900.

To investigate the functional relationships between proteins, protein-protein interaction networks of U1Cs were constructed using the STRING website. In this work, three representative U1C protein sequences of humans, mice and *Saccharomyces cerevisiae* (yeast) were chosen to generate interaction networks based on experiments and databases (Figure 3). With a confidence cutoff of 0.900, the resulting networks of human, mouse and yeast U1Cs contained 25, 15 and 35 functional partners, respectively (Figure 3 and Supplementary Table S3). As expected, overwhelming (besides ICP55 highlighted in green) predicted protein interactors of human, mouse and yeast U1Cs play important roles in pre-mRNA splicing. Interestingly, previous study has been demonstrated that PHF5A, partner of human U1C, had a contribution to colon carcinogenesis (Wang et al., 2019). Moreover, U1C may play a role in immune diseases (Satoh et al., 1996) (Pichlmair et al., 2011). However, specific interaction studies and functional verification require further analysis between U1Cs and their predicted protein partners. (Rsel et al., 2011)

### Analysis of Gene Structure and cDNA Conserved Motifs

To investigate the correlation between the genetic structural characteristics and potential function of the animal *U1C* gene family, it is necessary to compare the gene structure and analyse the presence of conserved cDNA motifs. Accordingly, their genomic organization and corresponding predicted conserved motifs were attached to the vertical phylogenetic tree (Supplementary Figure S6). To visualize full gene structures, the sequence of each *U1C* gene with the longest coding sequence (CDS) was used to reconstruct the exon-intron organization (Supplementary Figure S6, middle panels). Exons of *U1C* genes varied in number from one to seven, which suggested a large difference in gene structures of *U1C*s. Forty-eight sequences out of 110 *U1C* family genes had a seven exon-6 intron gene structure layout, accounting for 35.5% of the total number of members; 23 members had two exon-1 intron organizations, while 18 sequences did not contain any intron sequences (Supplementary Figure S6, middle panels). Moreover, only four *U1C* genes from *Rattus norvegicus* (ENSRNOP00000000586.6), *X. tropicalis* (ENSXETP00000053216.1), *Danio rerio* (ENSONIP00000018579.1) and *Takifugu rubripes* (ENSTRUP00000055830.1) have an extra exon that was not a coding exon. Usually, sequences clustered in the same subclade have similar exon-intron structures, such as six members from fish (light blue). Furthermore, sequences from one species may have different gene structures; for example, two sequences from *S. harrisii* were found, where one has one exon (ENSSHAP00000003382.1) and the other contains seven exons (ENSSHAP00000001057.1). Overall, differences of exon-intron distribution patterns were found in all four branches, indicating gene structural alterations may be involved in the evolution of this gene family among general animal phylogeny. On the other hand, genomic structural patterns show a high degree of similarity in the same subbranch, supporting the close evolutionary relationships of U1C in the same subbranch.

Due to relatively large differences in gene structures among U1C genes, MEME analysis was performed to determine whether motif composition in their cDNA sequences reflects any differences. Finally, the 10 most conserved motifs were identified based on the cDNA sequences of *U1Cs* using the web-server tool for motif analysis, MEME (Supplementary Figure S6, right panel). Broadly, more than half (67/110) of the *U1C* sequences contained ten conserved motifs. The positions and numbers of motifs of most animal *U1C* genes presented little difference among placentals (purple), marsupials, monotremes and reptiles (pink). In contrast, other species at the basal part of the tree, including Bpp0083134 (*Drosophila melanogaster*), F08B4.7 (*Caenorhabditis elegans*), YLR298C_mRNA (*S. cerevisiae*) and ENSCINP00000035879.1 (*Ciona intestinalis*), displayed low conservation in terms of motif composition, suggesting some degree of divergence in functions. Interestingly, no correlation was found between gene structures and conserved motifs. For example, sequences of ENSMMUP00000050488.1 (*Macaca mulatta*) with one exon, ENSRBIP00000024910.1 (*Rhinopithecus bieti*) with two exons, ENSPTRP00000071198.1 (*Pan troglodytes*) with three exons, ENSDNOP00000021117.1 (*Dasypus novemcinctus*) with 5 exons, ENSOARP00000011602.1 (*Ovis aries*) with 6 exons and ENSSSCP00000055297.1 (*Sus scrofa*) with seven exons all contained the ten identified conserved motifs (Supplementary Figure S6 and Supplementary Table S2). To conclude, comparing the conserved motifs at RNA/cDNA and protein level revealed that there are little discrepancies in codon usage of these orthologues, the similarity of the numbers and the positions of the motifs suggest conservation of animal U1C between different proteins and cDNAs. Furthermore, the comparison of cDNAs suggested that conserved motifs are not discovered in untranslated region, an area enriched in regulatory elements, giving additional information of conserved regulatory mechanism among these U1Cs.

### Transcript Isoforms and Conserved Splice Site Analysis

To study splicing patterns and conserved splice sites of animal U1C family genes, alternative splicing analysis among animal *U1C* genes was carried out. A total of 61 transcript isoforms from 26 *U1C* genes were obtained from the Ensembl database and linked to the phylogenetic relationships among selected species (Supplementary Figure S7 and 4). Nineteen *U1C* genes contained two transcript isoforms, five others contained three isoforms, and two contained four isoforms. Furthermore, MEME identified conserved protein motifs from potential protein products from the above transcript isoforms (Supplementary Figure S7, right panel). As expected, the primary transcript possesses the longest peptide sequence and most conserved motifs, while other alternative transcripts have shorter protein lengths and contain a reduced number of motifs. Furthermore, alternative first exons (AFEs) and alternative last exons (ALEs) are prominent AS events in U1Cs. Moreover, other splicing events, such as exon skipping, were also observed in *Oryctolagus cuniculus* and *Bos taurus*.

**FIGURE 4:**
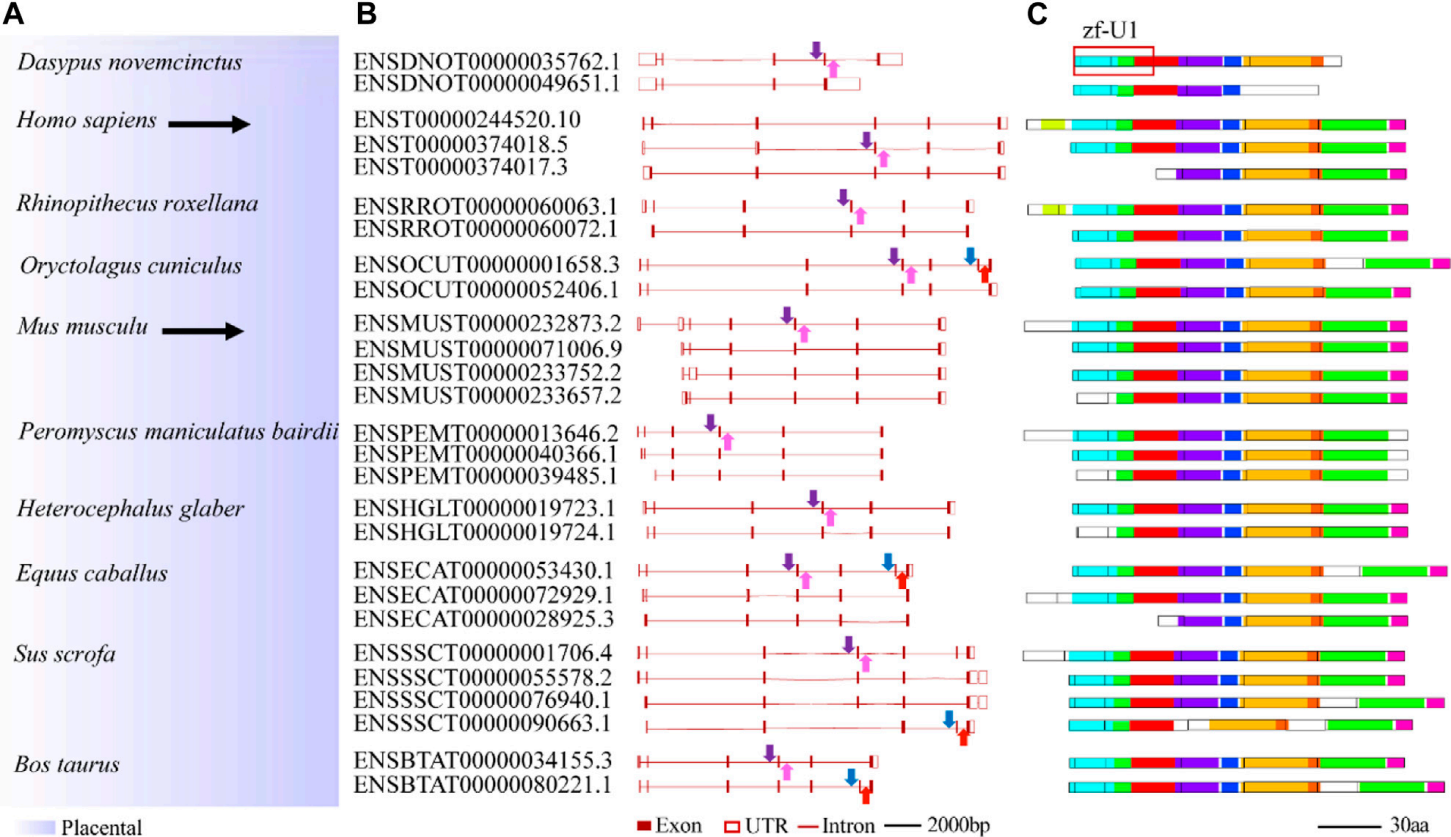
Summary of splicing isoforms for representative animal *U1C* genes. Transcript isoforms with transcript ID from 10 representative animal *U1C* genes are summarized on the **(A)**. And their corresponding gene structures redrawn based on online ensembl were displayed on the **(B)**. Conserved protein motifs of potential protein products from splicing isoforms are illustrated **(C)** with additional annotation to define exon-exon boundaries (black lines between boxes). Solid arrows with different colors represent different conserved splice sites or conserved sequences found in corresponding transcripts but without the detection of particular splicing events. For details on the ten motifs, refer to Supplementary Figure S7. U1C sequences from human and mouse are highlighted by black arrow. Marked red frame on the right corner of the figure shows overlapped region between protein domain (zf-U1) of U1C and conserved motifs.

**FIGURE 5:**
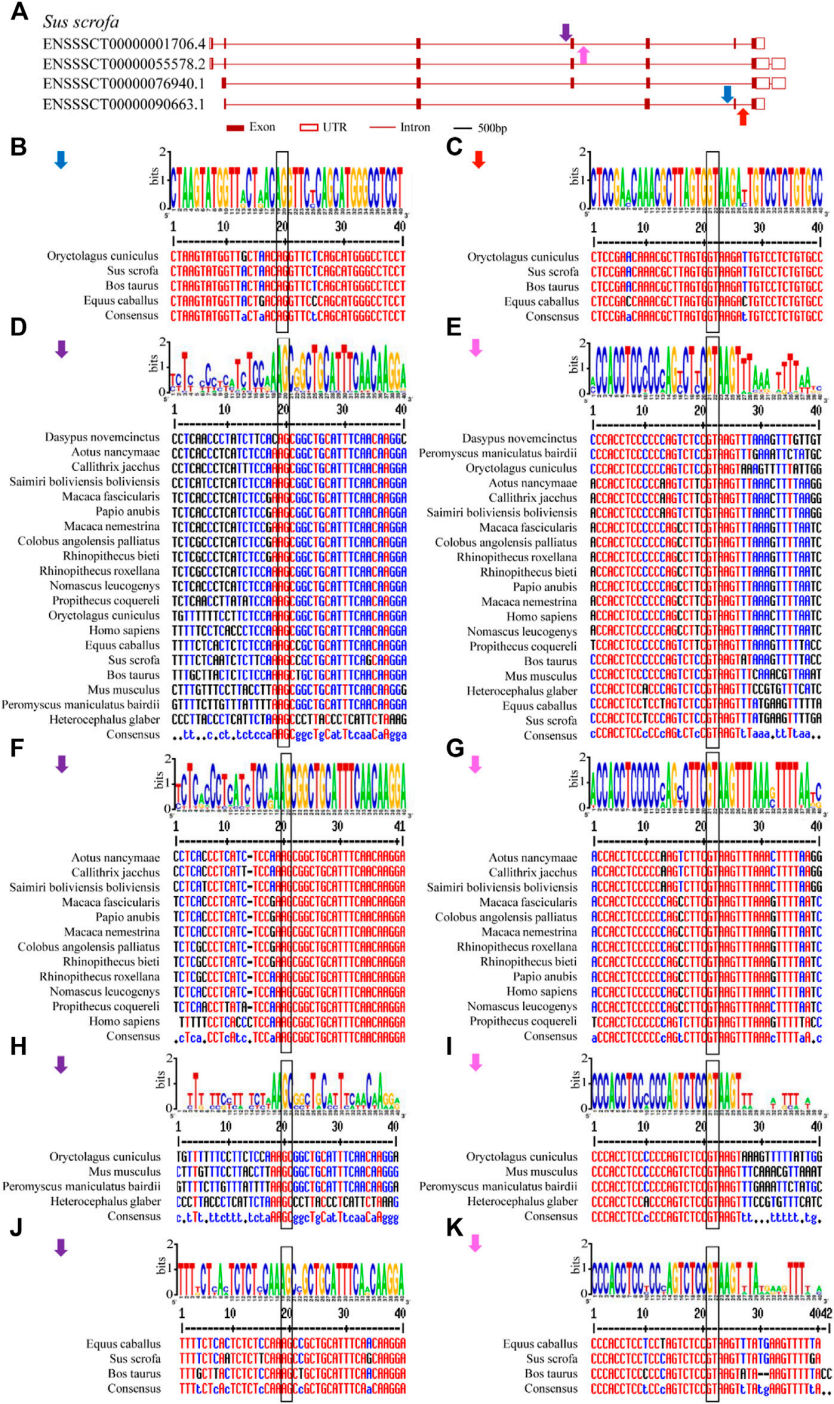
Conserved alternative splice site sequences analysis for animal *U1C* genes. Flanking sequences (40-bp) at conserved exon-intron boundaries of animal *U1C* genes are analyzed to show their consensus by online Weblogo and multiple alignment. The conventional 5’-GT or 3’-AG splice site dinucleotides are marked in black rectangle. **(A)** Four conserved splice sites found among animal *U1C* gene (*Sus scrofa*) were marked in blue arrow (type 1), red arrow (type 2), purple arrow (type 3) and pink arrow (type 4). **(B-E)** represent graph representation and multiple alignment of type 1, type 2**,** type 3 and type 4 conserved splice sites. **(F, H, J)** represent detailed analysis of type 3 splice site among sector “primates”, “rodents and lagomorphs” and “Other Mammals”. **(G, I, K)** represent detailed analysis of type 4 splice site among sector “primates”, “rodents and lagomorphs” and “Other Mammals”. The label for the y-axis is “bits”. Red, blue and black of multiple sequence alignments represent high consensus (default value: 90%), low consensus (default value: 50–90%) and neutral, respectively.

Conserved splice sites or conserved sequences were further identified. Four representative splice sites (Figure 5A) were identified by using a 40-bp flanking sequence at exon-intron junctions (Figure 4, Figure 5). Specifically, the 3’ splice site (marked by the blue arrow) and 5’ splice site (marked by the red arrow) of exon skipping events displayed high conservation in *O. cuniculus*, *S. scrofa*, *B. taurus* and *Equus caballus* (Figures 5B,C). Furthermore, type 3 and type 4 conserved splice sites (marked with purple and pink arrows, respectively) were found in the placentals, including primates, rodents/lagomorphs, and other mammals (Figures 5D,E). In detail, type 3 was conserved in “primates” (Figure 5F) and “other mammals” (Figure 5J), while type 4 was conserved in “primates” (Figure 5G), “rodents and lagomorphs” (Figure 5I) and “other mammals” (Figure 5K). In summary, four conserved splice sites were all located at the C-terminus of animal U1C. Conservation of these splice sites are dependent on animal subcategory, suggesting that this gene family may have evolved different splicing regulatory mechanisms within each animal subcategory.

### Expression Profile of Animal U1Cs

To further investigate the potential functions and regulatory mechanisms of animal *U1C*s in response to developmental cues or disease correlations, the expression patterns of model human and mouse (Ensg00000124562 and Ensmusg00000024217) *U1Cs* were analysed. Using the BAR HeatMapper Plus tool, expression data based on baseline studies from the online Expression Atlas were used to reconstruct a variety of biological aspects from expression profiles, such as developmental stage, different tissue and cell types, and disease conditions (Supplementary Figures S8–S13). First, the human *U1C* gene was highly expressed in several cancers, including B-cell non-Hodgkin lymphoma, breast adenocarcinoma, cervical adenocarcinoma, cervical squamous cell carcinoma, endometrial adenocarcinoma, esophageal adenocarcinoma, glioblastoma multiforme, lymphoma, melanoma, ovarian adenocarcinoma and squamous cell lung carcinoma (Supplementary Figure S8). Moreover, the human *U1C* gene was found to be expressed at a relatively high level in EBV-transformed lymphocyte, testis and brain tissues, such as the basal ganglion, cerebellum, cerebral cortex, hindbrain without cerebellum, midbrain, pituitary and diencephalon, and telencephalon (Supplementary Figure S11). Interestingly, it was also found that *U1C* accumulated in the mouse cerebellum (Supplementary Figure S10). The mouse *U1C* gene was found to be highly expressed in the ileum, ovary, spleen, thymus, uterus and muscle of leg (Supplementary Figure S10). Cell-type expression analysis showed that human *U1C* accumulated in common lymphoid progenitor, erythroblast and megakaryocyte-erythroid progenitor cells, while mouse *U1C* accumulated in hematopoietic stem cells and multipotent progenitor cells (Supplementary Figure S11 and Supplementary Figure S12). It was also observed that mouse *U1C* maintained higher expression levels at various sampling time points, in different strains, and with respect to developmental stages and somite stages than in various cell types (Supplementary Figure S12 and Supplementary Figure S13).

**FIGURE 6:**
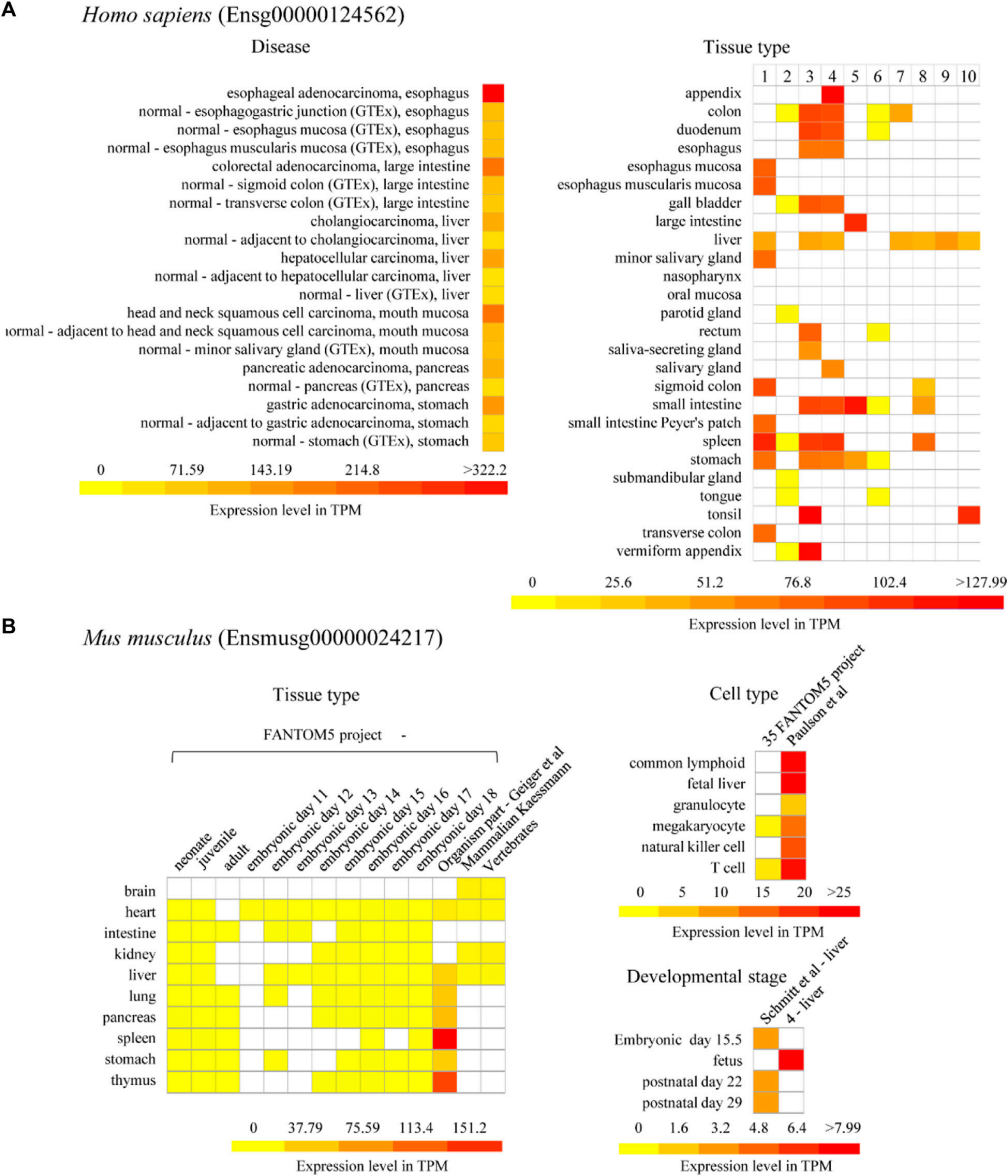
Expression of *U1C* in model organism *Homo sapiens* and *Mus musculus*. **(A)** Selected representative expression profile of human *U1C* gene (Ensg00000124562) related to human diseases and organism part is presented as heatmaps. Disease database is from The International Cancer Genome project: Pan-Cancer Analysis of Whole Genomes (PCAWG). GTEx means Genotype-Tissue Expression (GTEx) Project. No.1–10 of human-tissue type represents 1) GTEx, 2) FANTOM5 project - adult, 3) Uhlen’s Lab, 4) Hallstrom et al., 2014—Organism part, 5) NIH Epigenomics Roadmap, 6) FANTOM5 project—fetal, 7) Illumina Body Map, 8) ENCODE (M. Snyder lab), 9) Mammalian Kaessmann, 10) Zhu et al., 2018—Organism part, respectively. **(B)** Representative expression profiles of mouse *U1C* gene (Ensmusg00000024217) related to tissue type, cell type and developmental stages are shown as heatmaps. The columns in the heatmaps show the available experiments in Expression Atlas. The rows in the heatmaps show experimental condition such as developmental stage, different tissue and cell type and disease condition. White box presents there is no data available. Baseline expression levels are in TPM (transcripts per million). References for each project refer to Supplementary Table S6.

On the other hand, we particularly focused on the expression of *U1C* genes in the developmental stage (Supplementary Figure S11 and Supplementary Figure S13) and in digestive diseases or digestive systems in this work (Figure 6). In detail, the human *U1C* gene in cancer tissue had a higher expression level than in normal paracarcinoma tissue and normal tissue (Figure 6A). In particular, the expression level of human *U1C* was highest in esophageal adenocarcinoma. Furthermore, according to multiple datasets, the human *U1C* gene had a relatively higher expression level in digestive organs, such as the large intestine, appendix, tonsil, spleen, and colon (Figure 6A). However, tissue-specific expression profiles from multiple datasets showed that the *U1C* gene maintained low expression levels in various digestive organs in mouse, including the intestine, liver, pancreas, spleen, stomach and small intestine (Figure 6B and Supplementary Figure S10). The development map indicated that the expression of the human *U1C* gene was highest in infants, followed by adults, and lowest in adolescents (Supplementary Figure S11). With regard to the developmental stages of mice, *U1C* preferably accumulated at the fetal stage, and its expression was higher than that at other developmental stages (Supplementary Figure S13). In conclusion, based on the above expression data analysis, we found that U1C is highly expressed in digestive diseases, which may be used as a valuable diagnostic or therapeutic protein target during clinical treatment. In addition, the human U1C gene has high expression level in digestive organs compared with the expression of its orthologue in mouse, the potential function of this gene associated with the organ development or diseases are worth doing further investigation.

## Discussion

### Assessment of Phylogenetic Relationships and Functional Conservation in Animal *U1C*s

As an important U1-specific snRNP, U1C has a direct influence on 5’ splice site recognition (Pichlmair et al., 2011). U1C interacts with other splicing factors, such as U1-70K, precedes pre-mRNA/U1 snRNA base pairing and is important in the early stages of the splicing pathway (Du and Rosbash, 2002) (Knoop and Baker, 2000). In the present study, we systematically identified 110 *U1C* genes from 61 animal species and reconstructed the phylogenetic relationships of these selected genes. Clear topology showed that U1C proteins can be broadly divided into four groups including placentals, marsupials, monotremes and reptiles, along with other species, which correlates well with the evolution of the animal lineage (Figure 1, Supplementary Figure S2 and Supplementary Figure S4 (left panel)). Moreover, although over 65% (72/110) of these genes contain multiple copies of the *U1C* gene (Supplementary Table S1), analysis of protein structures and motifs of cDNAs and protein domains suggested that this gene family maintains conserved functions, indicating their functional redundancy (Supplementary Figure S2 and Supplementary Figure S5). Furthermore, the conserved splicing pattern of animal U1Cs showed that the majority of transcript isoforms of animal *U1Cs* tended to form proteoforms with N-terminal truncation (Figure 4). However, the truncation of the N-terminus does not seem to cause the lack or truncation of the main protein domain (zf-U1), which is required for the U1C protein to bind to the U1 snRNP particle (Klein Gunnewiek et al., 1995), suggesting that U1C proteins may preserve conserved function. This functional conservation is a result of evolutionary relationships between genes, and it has been demonstrated that the zinc finger motif is sufficient for the splice site recognition function in most cases. Consensus splice site sequences and exon synteny analysis showed that AFE and ALE are prominent AS events in animal U1Cs. The transcript isoforms of animal U1Cs have similar gene structure, which may indicate that they could have similar functions in regulating gene expression and interacting protein partners. On the other hand, all protein isoforms are generally considered functional; thus, the different biological functions of animal U1C protein isoforms require further experimental studies. For example, a plant truncated Calcium-dependent Protein kinase 28 (CPK28) was demonstrated to negatively regulate immune responses (DressanoWeckwerth et al., 2020). As shown in Figure 3, the human U1C protein had an N-terminal truncated isoform without the zf-U1 domain, which may have certain biological functions and necessitates further study. Moreover, an exon skipping event was found in vertebrates according to conserved alternative splice site analysis (Figure 5 and Supplementary Figure S7), and its mechanism of action also needs to be further investigated and experimentally validated.

### Functional Diversification of Animal *U1Cs* Based on Their Expression Profiles

The U1 snRNP-specific U1C protein is a pivotal component for 5’ splice site recognition and during early spliceosome assembly. Previous studies have demonstrated that U1 snRNP and U1-70K are involved in Alzheimer’s disease (Bai et al., 2013). However, few studies have elucidated the link between human disease and U1C. Therefore, visualized analysis from publicly available microarray expression data in the Expression Atlas would provide valuable information on their potential function during the developmental processes of animals or to characterize disease-related factors. Here, according to publicly available data, we found that human *U1C* accumulated in ovarian adenocarcinoma, esophageal adenocarcinoma, etc., which would provide a novel target and orientation for anticancer drug development (Chen et al., 2019). Meanwhile, based on available proteomics datasets (Tyanova et al., 2016) (Zhang et al., 2014), high expression of *U1C* was observed in breast, colon and rectal cancers, suggesting its potential involvement and functional role in the development of cancer in these organs (Figure 6 and Supplementary Figure S8). Interestingly, human *U1C* was highly expressed in the testis, and mouse *U1C* was highly expressed in the ovary, indicating that animal U1Cs may serve a potential role in animal fertility. Furthermore, expression data showed that *U1Cs* from humans and mice displayed different expression patterns across different cell types, organs and developmental stages (Supplementary Figure S8–S13), indicating species- and tissue-specific regulation at the transcription and translational levels. In addition, because current expression data are mainly focused at the transcriptional level or full-length protein level, the expression profiles of *U1Cs* at the isoform level have not been studied in detail. Hence, it is necessary to further investigate the expression profiles of animal U1C isoforms by quantitative real-time PCR with isoform-specific primers or SWATH-MS (sequential window acquisition of all theoretical mass spectra)-based quantitative approaches (Zhu et al., 2018) (Zhu et al., 2017). We have a successful case in plants about how to locate a potential stress-related plant SRP (splicing-related protein) and how to produce a follow-up experimental design to study its biological function, which would provide a reference for us to further investigate the specific function of each *U1C* transcript isoform (Chen et al., 2021).

### Comparison of U1Cs in Animals, Yeast and Plants

Interestingly, splicing machinery is not exactly similar among humans, yeast and Arabidopsis. In particular, although the genomic structures of human and plant U1Cs are similar (Supplementary Figure S14A), different domains/conserved amino acid sequences are observed at their C-terminal regions (Supplementary Figure S14B). This suggests that U1C proteins are conserved at the N-terminus and even at splice sites located between exon 1 and exon 2 (Supplementary Figure S14B,C). Thus, functional diversification is potentially mediated by the C-terminal regions.

## Conclusion

In this study, we identified a total of 110 *U1C* genes from 61 animal species and comprehensively analysed their phylogenetic relationships, genomic organization, motif and protein domain enrichment and splicing pattern conservation, providing a foundation for molecular research on U1C proteins with respect to their roles in human diseases investigated in mammalian cell lines or animal models.

## Data Availability

The datasets presented in this study can be found in online repositories. The names of the repository/repositories and accession number(s) can be found in the article/Supplementary Material.
