# Supplementary material for: Phylogenetic Comparison and Splicing Analysis of the U1 snRNP-specific Protein U1C in Eukaryotes
Source: Front Mol Biosci. 2021 Sep 9;8:696319. doi: 10.3389/fmolb.2021.696319 (PMC8458698; doi:10.3389/fmolb.2021.696319)
Supplement: Supplementary file 1 [file DataSheet1.zip › Additional/2020-06 S Figures -revised.docx]

**
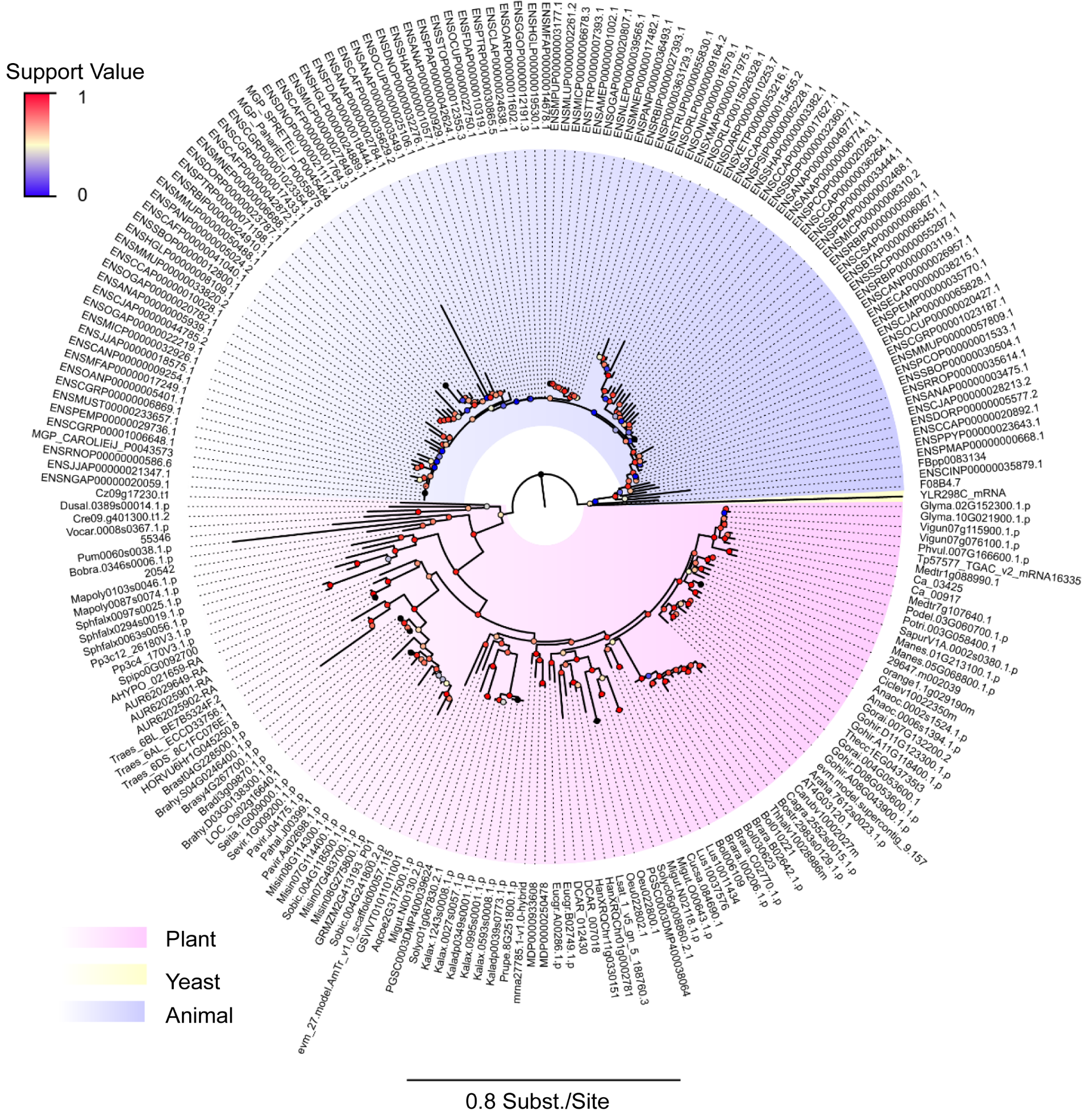
**

**Figure S1 Phylogenetic comparison of *U1C*s among plants, yeast and animals.** The circle phylogenetic tree of 110 U1C proteins from 61 animals and 114 U1C proteins from 72 plants was constructed based on maximum-likelihood by using PhyML v3.037. Bootstrap values are labeled as color gradient at each branch point (0-1). Species from plant, yeast and animal are represented with pink, yellow and blue gradient colors, respectively. Detailed information of all *U1C* genes is shown in Table S1 and Table S5.


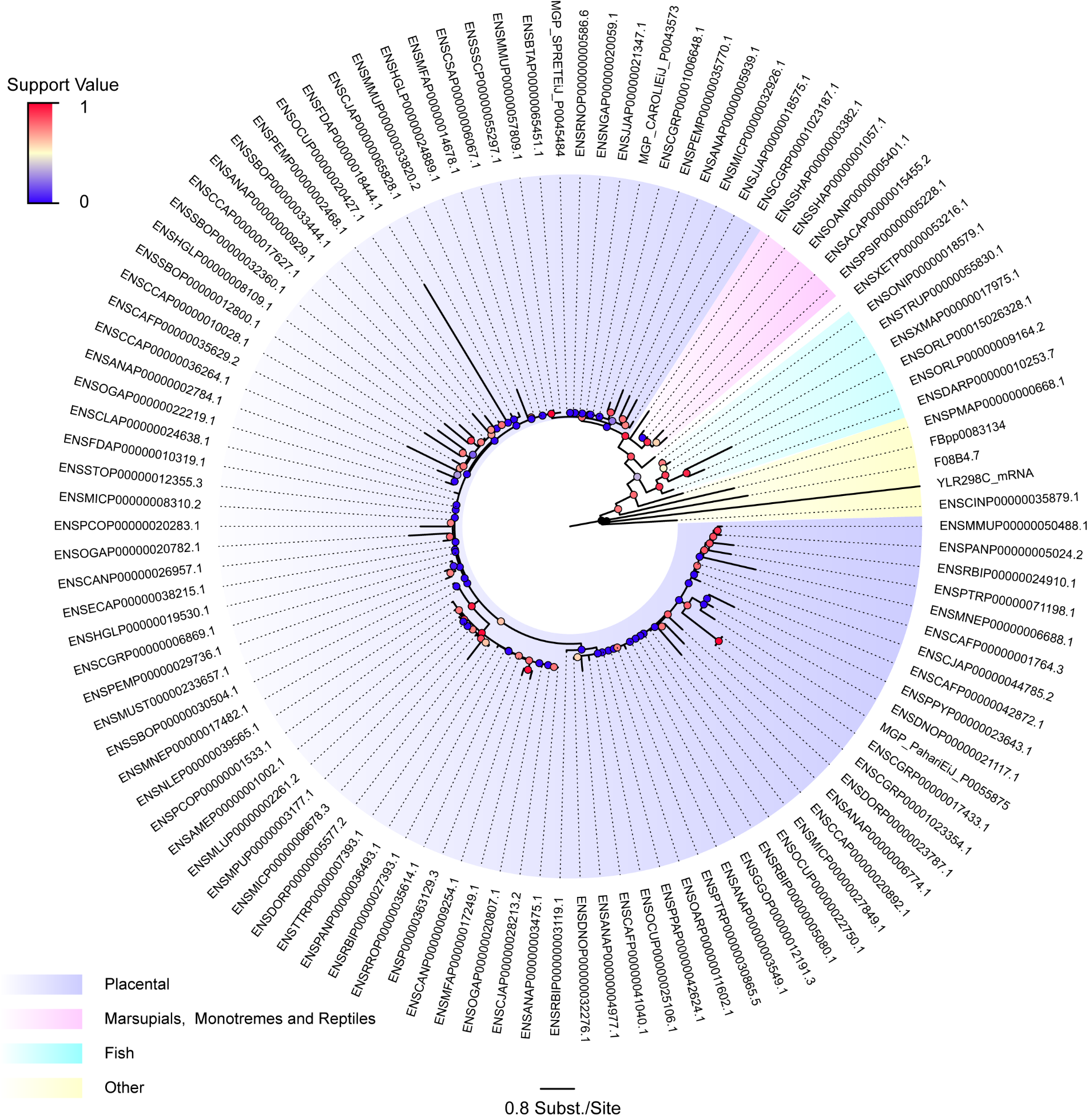


**Figure S2 Circle phylogenetic analysis of the *U1C* gene family in animals.** The circle phylogenetic tree of 110 U1C proteins from 61 animals was constructed based on maximum-likelihood by using PhyML v3.037. Bootstrap values are labeled as color gradient at each branch point (0-1). Species from different taxonomies are represented with different gradient colors. Detailed information of all *U1C* genes is shown in Table S1.


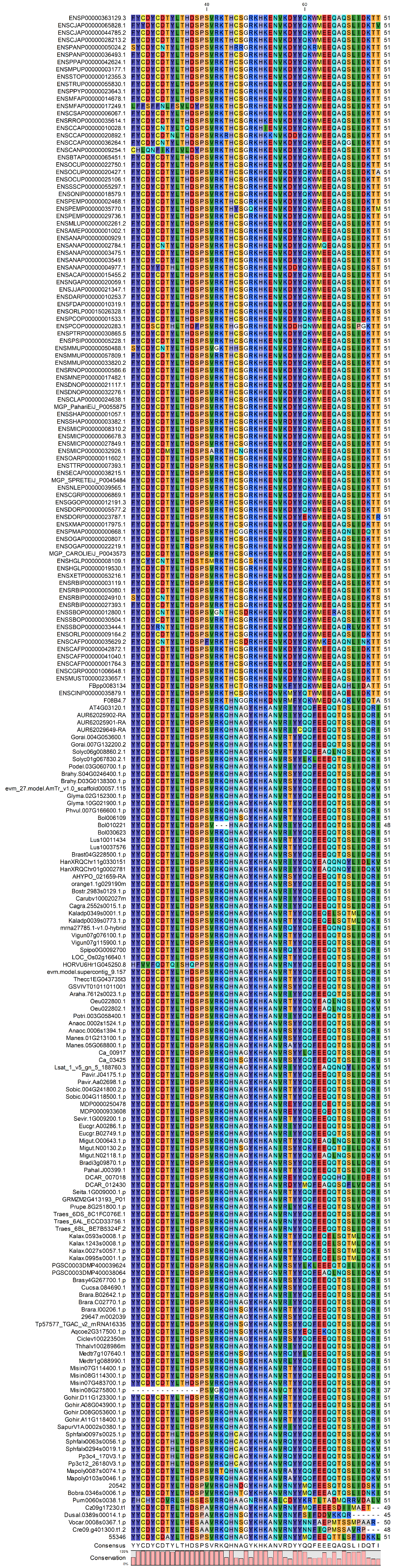


**Figure S3 Multiple sequence alignment of animal and plant *U1C* sequences used for the conservative analysis.**

**
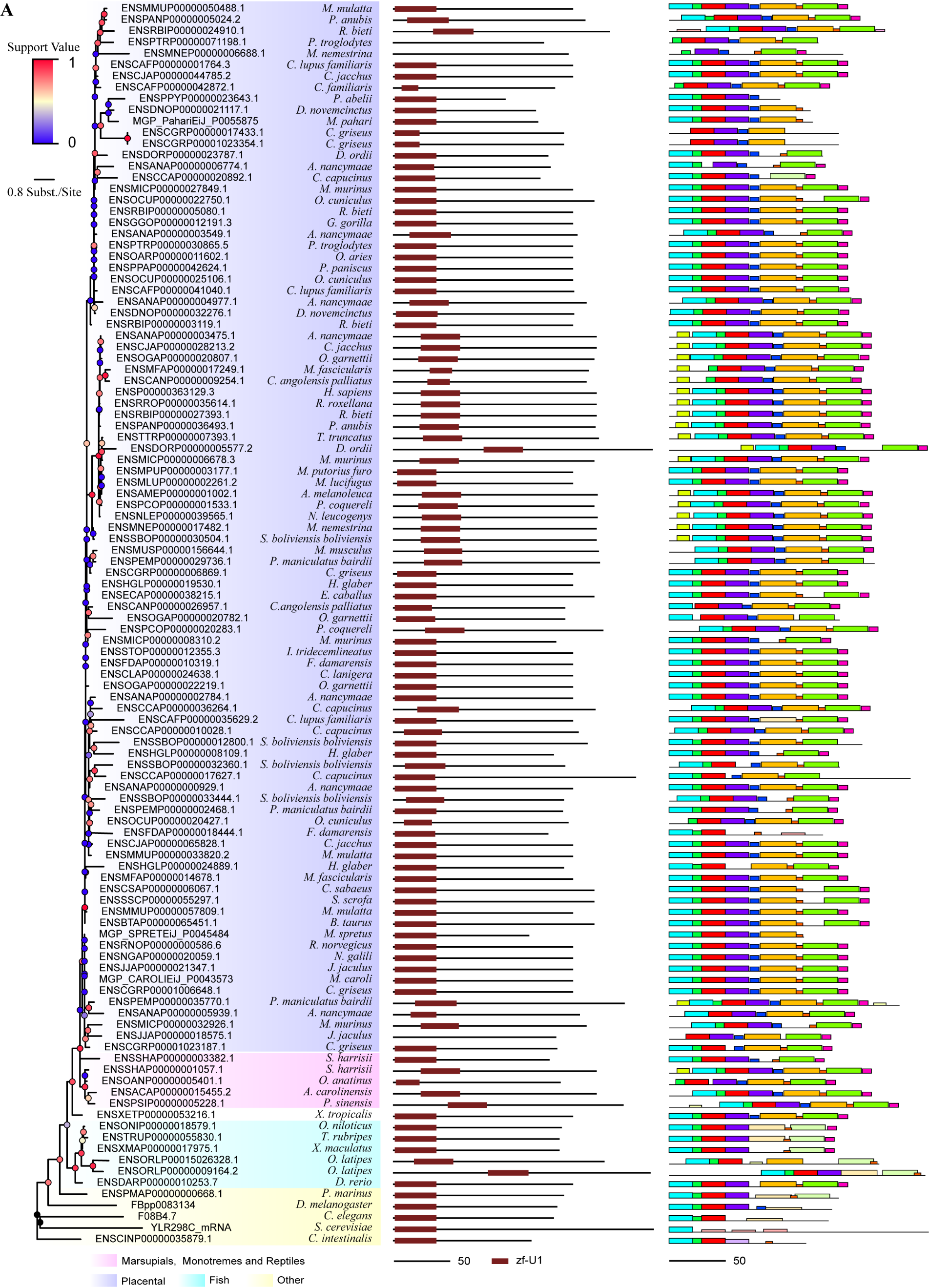
**


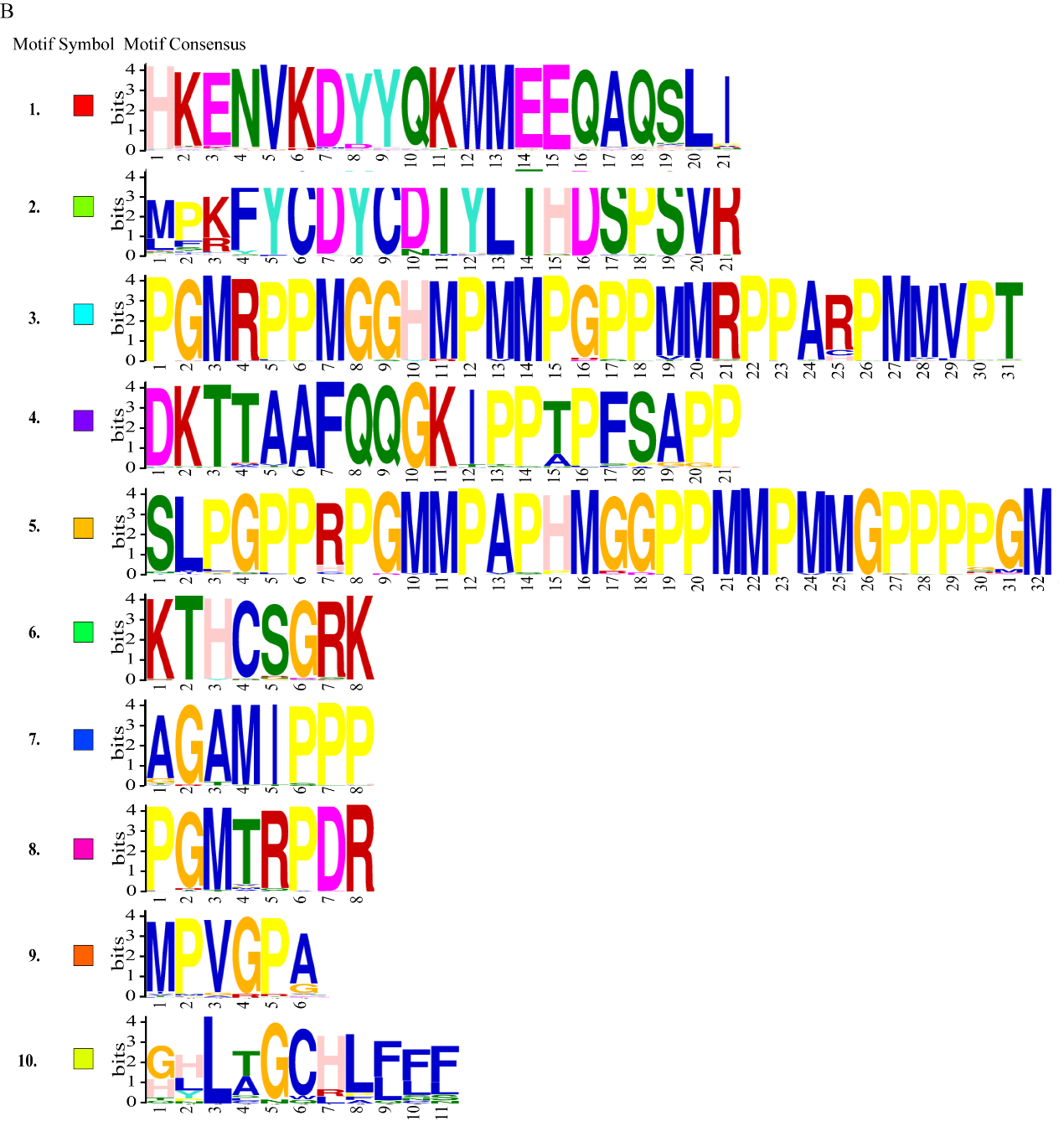


Figure S4 continued.

**Figure S4 Comparisons of structure and motif analysis of animal U1C proteins. (A)** The vertical phylogenetic tree is listed on the left panel. The legend on the top left corner represents support value of the tree. Blue/0 to red/1 means the range of values. The length of the scale bar corresponds to 0.8 substitutions per site. The predicted protein domains (zf-U1) by online software HMMER are listed on the middle panel. The identified protein conserved motifs with MEME analysis are shown on the right panel. The 10 most frequently discovered motifs are shown in different solid colors and additional scanned sites (p-values less than 0.0001) are shown in transparent color. **(B)** The logos and detailed information of ten motifs from MEME are shown below. The height of symbols indicates the degree frequency of each amino acid at that position. The label for the y-axis is "bits". U1C sequences from human, mouse and *C. elegens* and yeast are highlighted by red arrow.


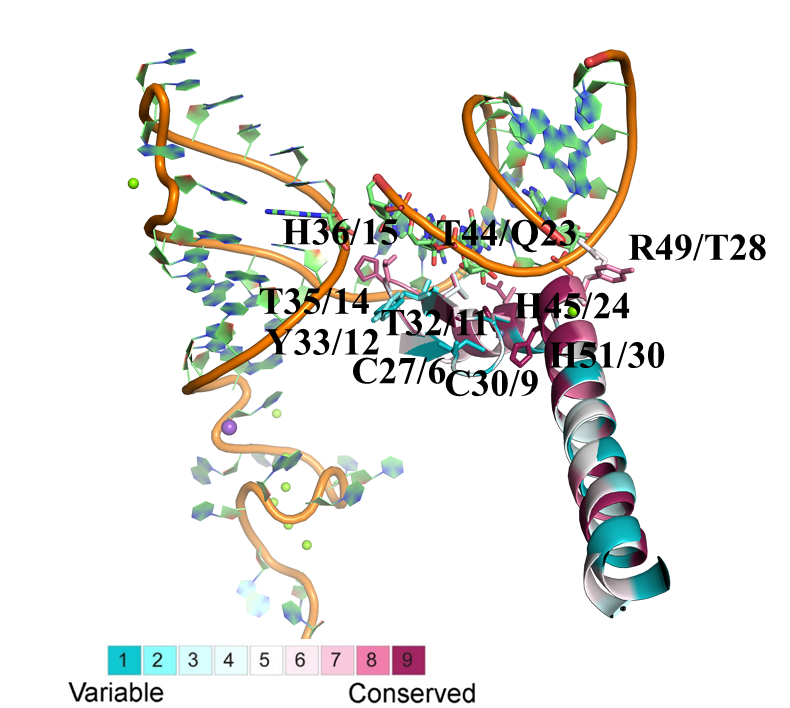


**Figure S5 The comparison of evolutionary conservation between animal and plant U1Cs.** The ribbon representation is colored according to ConSurf Grade (1-blue to 9-purple) by using identified protein sequences of animal/plant U1Cs. The residue numbers of human/*Arabidopsis* (At4g03120) are labeled in the figure. The sequences used here were shown in Figure S3. C27/6, C30/9, H36/15, H45/24, H51/30, R49/T28, T32/11, T35/14 and Y33/12 are residues of human/*Arabidopsis* U1Cs. C, H, R, T and Y are symbol of cysteine, histidine, arginine, threonine and tyrosine, respectively.


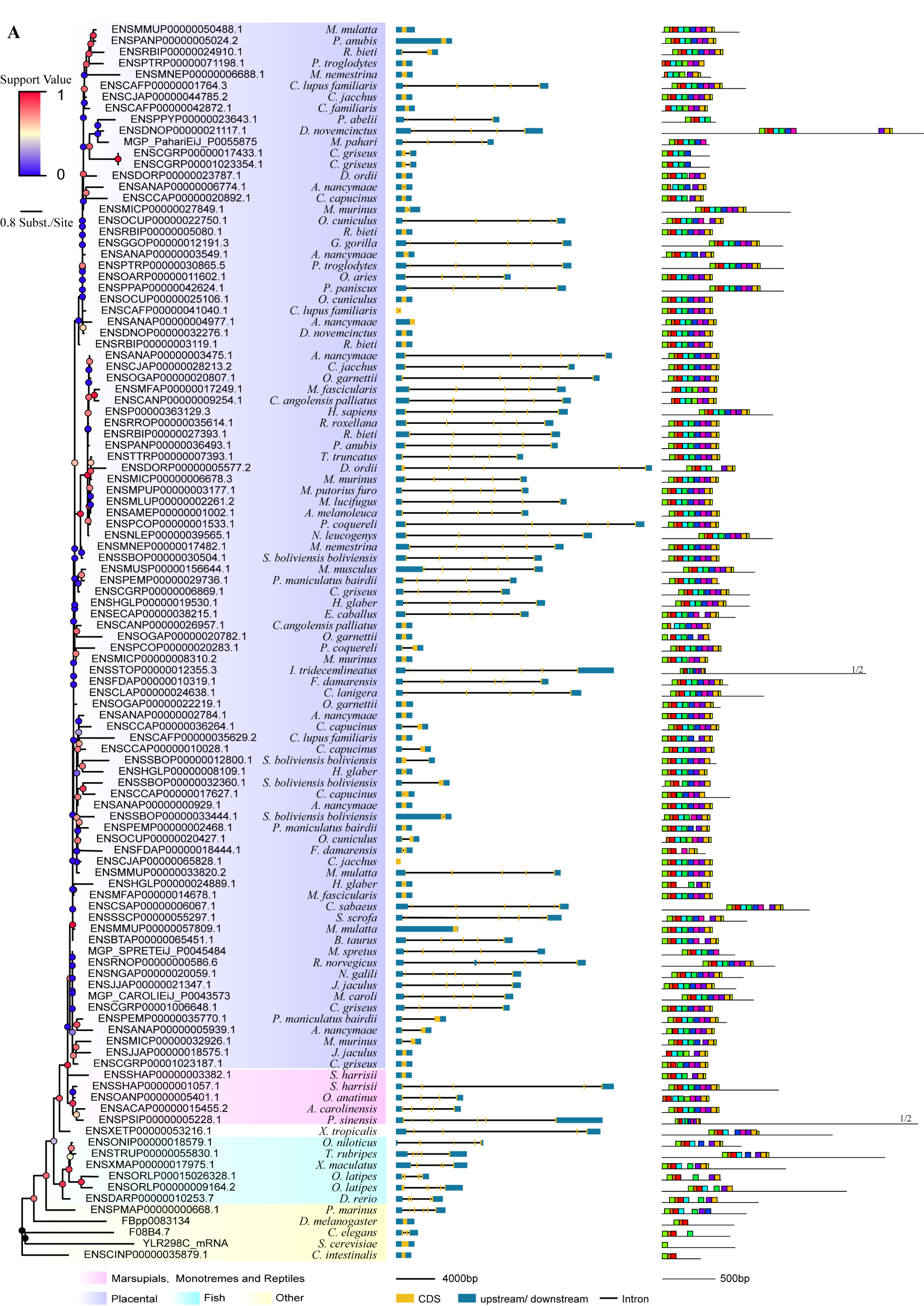

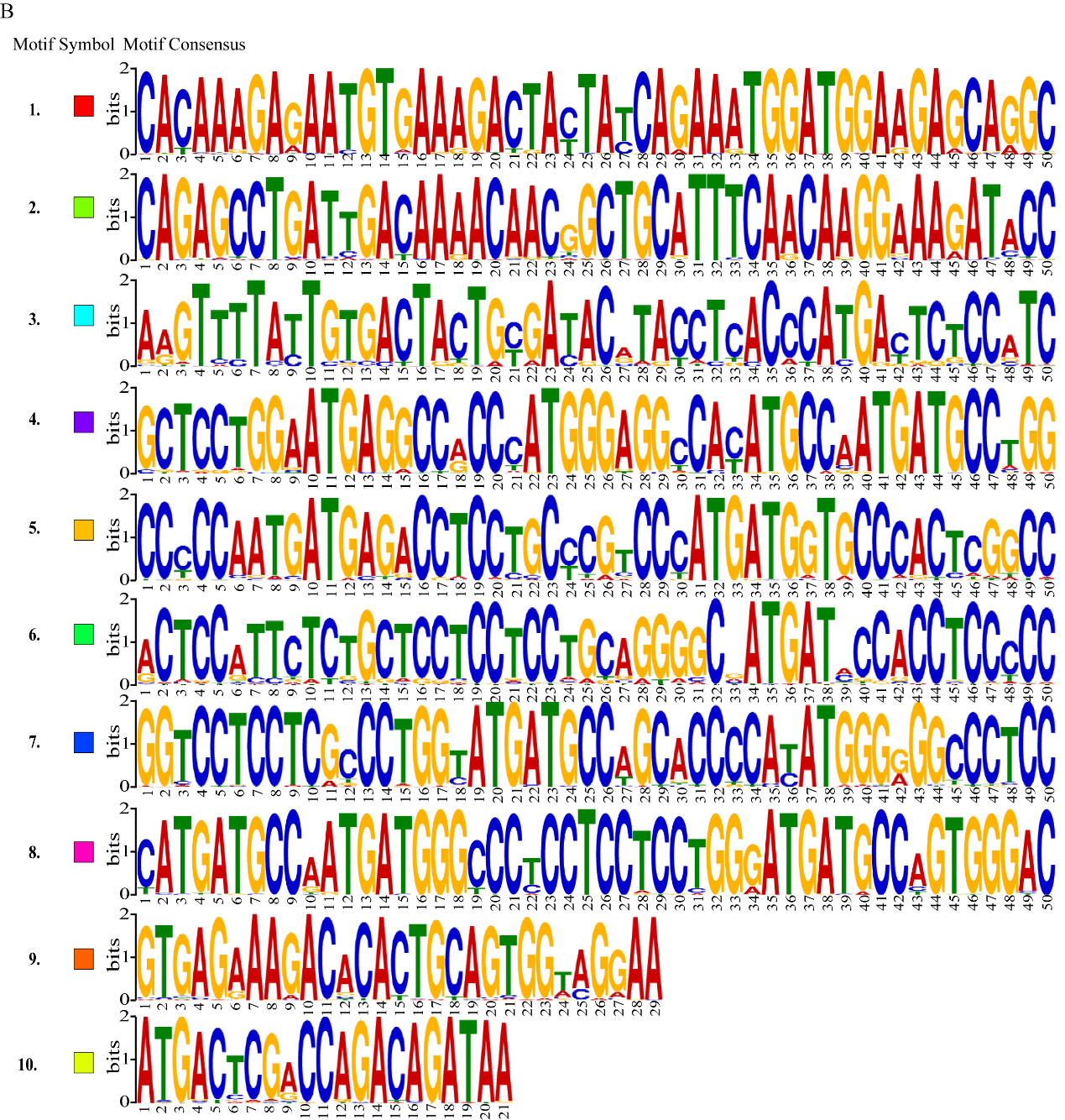


Figure S6 continued.

**Figure S6 Identification and comparison of genomic organization and conserved motifs among animal *U1C* genes.** **(A)** The vertical phylogenetic tree is listed on the left panel. The gene structure by GSDS2.0 website are listed on the middle panel. The identified cDNA conserved motifs with MEME analysis are shown on the right panel. The 10 most frequently discovered motifs are shown in different solid colors and additional scanned sites (p-values less than 0.0001) are shown in transparent color. **(B)** The logos and detailed information of ten motifs from MEME are shown below. The height of symbols indicates the degree frequency of each nucleic acid at that position. The label for the y-axis is "bits".


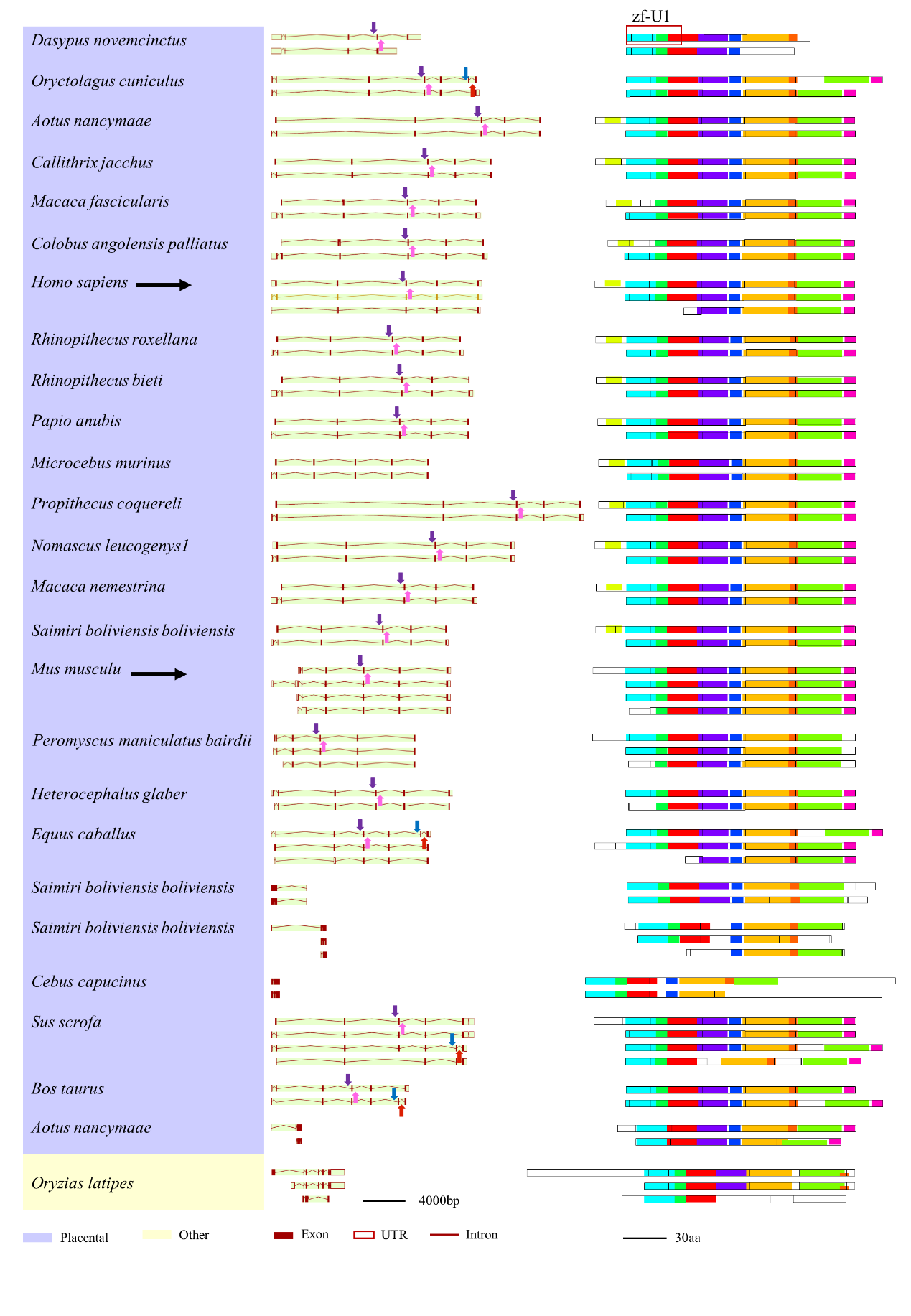


**Figure S7 Summary of splicing isoforms for animal *U1C* genes.** Transcript isoforms from 26 animal *U1C* genes are summarized on the left panel. And their corresponding gene structures were displayed on the middle panel. Conserved protein motifs of potential protein products from splicing isoforms are illustrated (right panel) with additional annotation to define exon-exon boundaries (black lines between boxes). Solid arrows with different colors represent different conserved splice sites or conserved sequences found in corresponding transcripts but without the detection of particular splicing events. For details on the ten motifs, refer to Figure 1. U1C sequences from human and mouse are highlighted by black arrow. Marked red frame on the right corner of the figure shows overlapped region between protein domain (zf-U1) and conserved motifs.


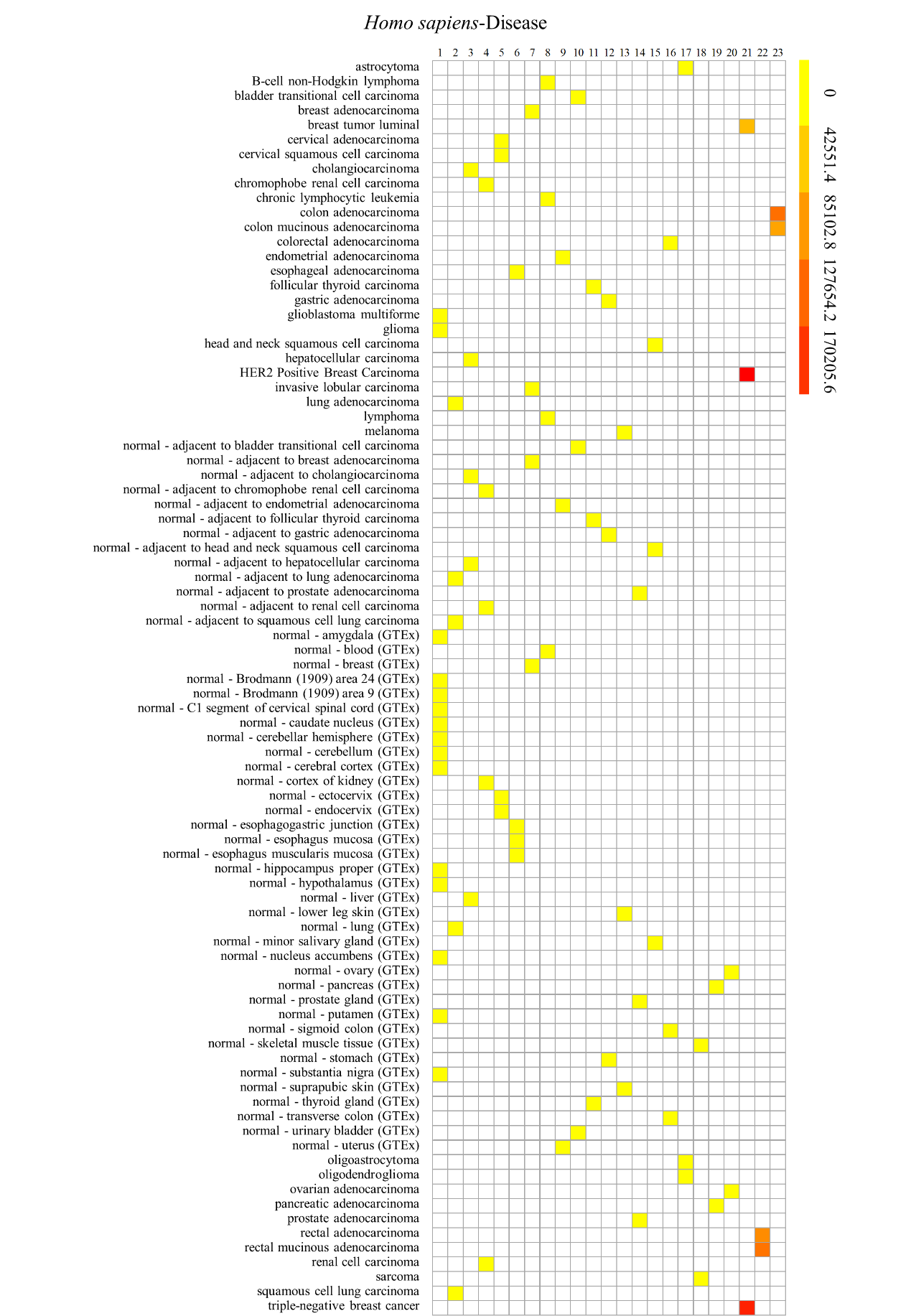


**Figure S8 Heatmap representation of disease expressions** **of *U1C* in *Homo sapiens*.** The columns in the heatmaps show the available experiments in Expression Atlas. The rows in the heatmaps show experimental condition such as developmental stage, different tissue and cell type and disease condition. White box presents there is no data available. No. 1-20 are from RNA-seq experiments and No. 21-23 are proteomics data. No.1-23 represent (1) Pan-Cancer Analysis of Whole Genomes - brain, (2)Pan-Cancer Analysis of Whole Genomes - lung, (3) Pan-Cancer Analysis of Whole Genomes - liver, (4) Pan-Cancer Analysis of Whole Genomes - kidney, (5) Pan-Cancer Analysis of Whole Genomes - uterine cervix, (6) Pan-Cancer Analysis of Whole Genomes - esophagus, (7) Pan-Cancer Analysis of Whole Genomes - breast, (8) Pan-Cancer Analysis of Whole Genomes - blood, (9) Pan-Cancer Analysis of Whole Genomes - uterus, (10) Pan-Cancer Analysis of Whole Genomes - urinary bladder, (11) Pan-Cancer Analysis of Whole Genomes - thyroid gland, (12) Pan-Cancer Analysis of Whole Genomes - stomach, (13) Pan-Cancer Analysis of Whole Genomes - skin, (14) Pan-Cancer Analysis of Whole Genomes - prostate gland, (15) Pan-Cancer Analysis of Whole Genomes - mouth mucosa, (16) Pan-Cancer Analysis of Whole Genomes - large intestine, (17) 3 Glioma subtypes, (18) Pan-Cancer Analysis of Whole Genomes - skeletal muscle tissue, (19) Pan-Cancer Analysis of Whole Genomes - pancreas, (20) Pan-Cancer Analysis of Whole Genomes - ovary, (21) Proteomics - Tissue - Breast cancer - Tyanova et al., (22) Proteomics - Tissue - Colon and Rectal cancer-rectum, (23) Proteomics - Tissue - Colon and Rectal cancer - colon, respectively. Baseline expression levels from RNA-seq experiments are in TPM (transcripts per million) and proteomics expression levels are mapped to yellow-red per experiment basis. References for each project refer to Table S6.


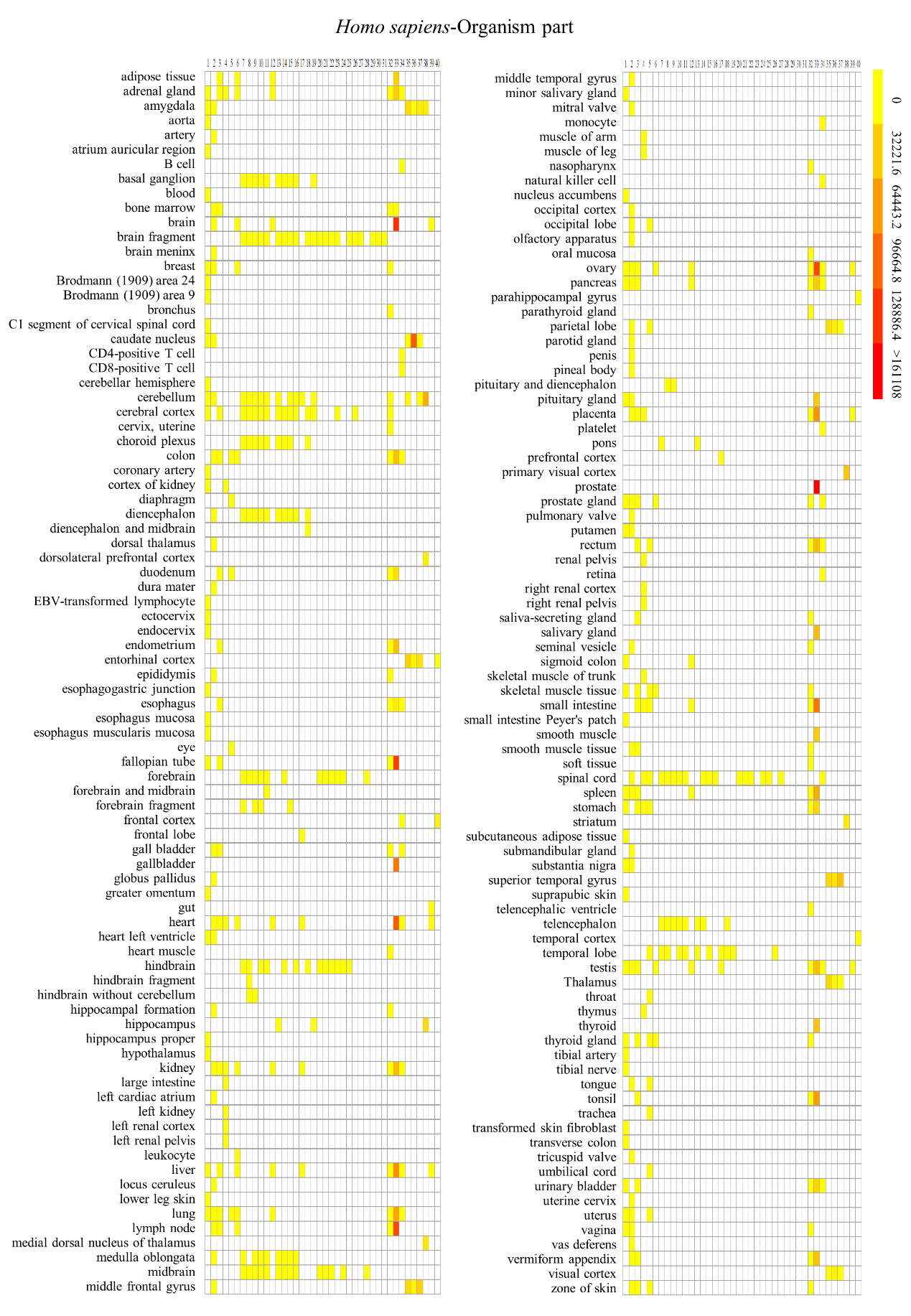


**Figure S9 Organism part** **expressions of human *U1C* gene.** The columns in the heatmaps show the available experiments in Expression Atlas. The rows in the heatmaps show experimental condition such as developmental stage, different tissue and cell type and disease condition. White box presents there is no data available. No. 1-31 are from RNA-seq experiments and No. 32-40 are proteomics data. No.1-40 represent (1) GTEx, (2) 68 FANTOM5 project - adult, (3) 32 Uhlen's Lab, (4) 19 NIH Epigenomics Roadmap, (5) 68 FANTOM5 project - fetal, (6) Illumina Body Map, (7) HDBR developing brain - 9 post conception weeks, (8) HDBR developing brain - 12 post conception weeks, (9) HDBR developing brain - Carnegie Stage 22, (10) HDBR developing brain - 11 post conception weeks, (11) HDBR developing brain - 10 post conception weeks, (12) ENCODE (M. Snyder lab), (13) HDBR developing brain - 16 post conception weeks, (14) HDBR developing brain - Carnegie Stage 23, (15) HDBR developing brain - 13 post conception weeks, (16) HDBR developing brain - Late 8 post conception weeks, (17) Mammalian Kaessmann, HDBR developing brain - 14 post conception weeks, (18) HDBR developing brain - 17 post conception weeks, (19) HDBR developing brain - Carnegie Stage 21, (20) HDBR developing brain - Carnegie Stage 18, (21) HDBR developing brain - Carnegie Stage 17, (22) HDBR developing brain - Carnegie Stage 20, (23) HDBR developing brain - Carnegie Stage 14, (24) HDBR developing brain - Carnegie Stage 19, (25) HDBR developing brain - 15 post conception weeks, (26) HDBR developing brain - Carnegie Stage 16, (27) HDBR developing brain - Carnegie Stage 13, (28) HDBR developing brain - Carnegie Stage 15, (29) HDBR developing brain - 20 post conception weeks, (30) HDBR developing brain - 19 post conception weeks, (31) Human Protein Atlas, Wang et al. 2019, (32) Human Proteome Map – adult, (33) brain regions in Alzheimer's McKetney et al. 2019 - Alzheimer's disease, (34) early braak stage, brain regions in Alzheimer's McKetney et al. 2019 - normal, (35) normal, (36) brain regions in Alzheimer's McKetney et al. 2019 - Alzheimer's disease, (37) late braak stage, brain regions, (38) Human Proteome Map - fetus, (39) brain regions in Alzheimer's MendonГѓВ§a et al. 2019 - normal, (40) normal, respretively. Baseline expression levels from RNA-seq experiments are in TPM (transcripts per million) and proteomics expression levels are mapped to yellow-red per experiment basis. References for each project refer to Table S6.


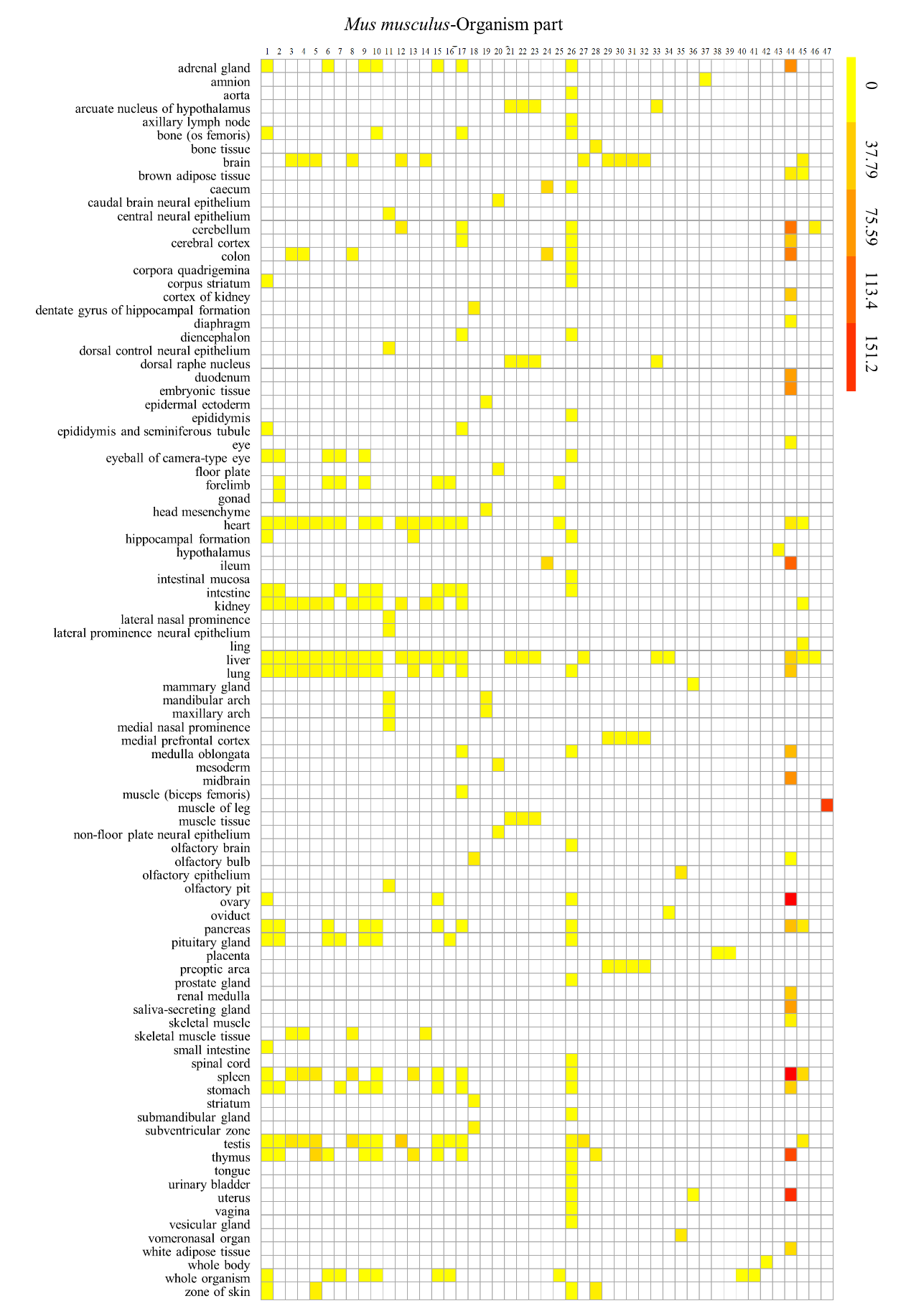
**Figure** **S10 Organism part expression of *U1C* in** ***Mus musculus*.** The columns in the heatmaps show the available experiments in Expression Atlas. The rows in the heatmaps show experimental condition such as developmental stage, different tissue and cell type and disease condition. White box presents there is no data available. No.1-47 represent (1) 49 FANTOM5 project - neonate, (2) 49 FANTOM5 project - embryonic day 15, (3) 9 in 3 strains - DBA/2J, (4) 9 in 3 strains - CD1, (5) 9, (6) 49 FANTOM5 project - embryonic day 14, (7) 49 FANTOM5 project - embryonic day 12, (8) 9 in 3 strains - C57BL/6, (9) 49 FANTOM5 project - embryonic day 17, (10) 49 FANTOM5 project - embryonic day 16, (11) 14 - embryonic day 10.5, (12) Mammalian Kaessmann, (13) 6, (14) Vertebrates, (15) 49 FANTOM5 project - embryonic day 18, (16) 49 FANTOM5 project - embryonic day 13, (17) 49 FANTOM5 project - juvenile, (18) 4, (19) 14 - embryonic day 9.5, (20) 14 - embryonic day 8.5, (21) 4 Bonthuis et al - CastEiJ, (22) 4 Bonthuis et al - (CastEiJ X C57BL/6J)F1, (23) 4 Bonthuis et al - (C57BL/6J X CastEiJ)F1, (24) Developing gut, (25) 49 FANTOM5 project - embryonic day 11, (26) 49 FANTOM5 project - adult, (27) 3 Soumillon et al, (28) 3, (29) Gregg et al - CAST/EiJ, (30) Gregg et al - C57BL/6J, (31) Gregg et al - (CAST/EiJ X C57BL/6J)F1, (32) Gregg et al - (C57BL/6J X CAST/EiJ)F1, (33) 4 Bonthuis et al - C57BL/6J, (34) 49 FANTOM5 project - pregnant adult day 1, (35) 2, (36) 49 FANTOM5 project - pregnant adult day 19, (37) 49 FANTOM5 project - pregnant adult day 17.5, (38) 49 FANTOM5 project - pregnant adult day 17, (39) 49 FANTOM5 project - pregnant adult day 10, (40) 49 FANTOM5 project - embryonic day 17.5, (41) 49 FANTOM5 project - embryonic day 14.5, (42) 49 FANTOM5 project - embryo, (43) 4 Bonthuis et al - Idaho derived wild mouse, (44) Organism part - Geiger et al, (45) Organism part - Huttlin et al, (46) Organism part - Meierhofer et al, (47) Skeletal muscle - Deshmukh et al - myotube, C2C12,, respectively. Baseline expression levels from RNA-seq experiments experiments are in TPM (transcripts per million). References for each project refer to Table S6.


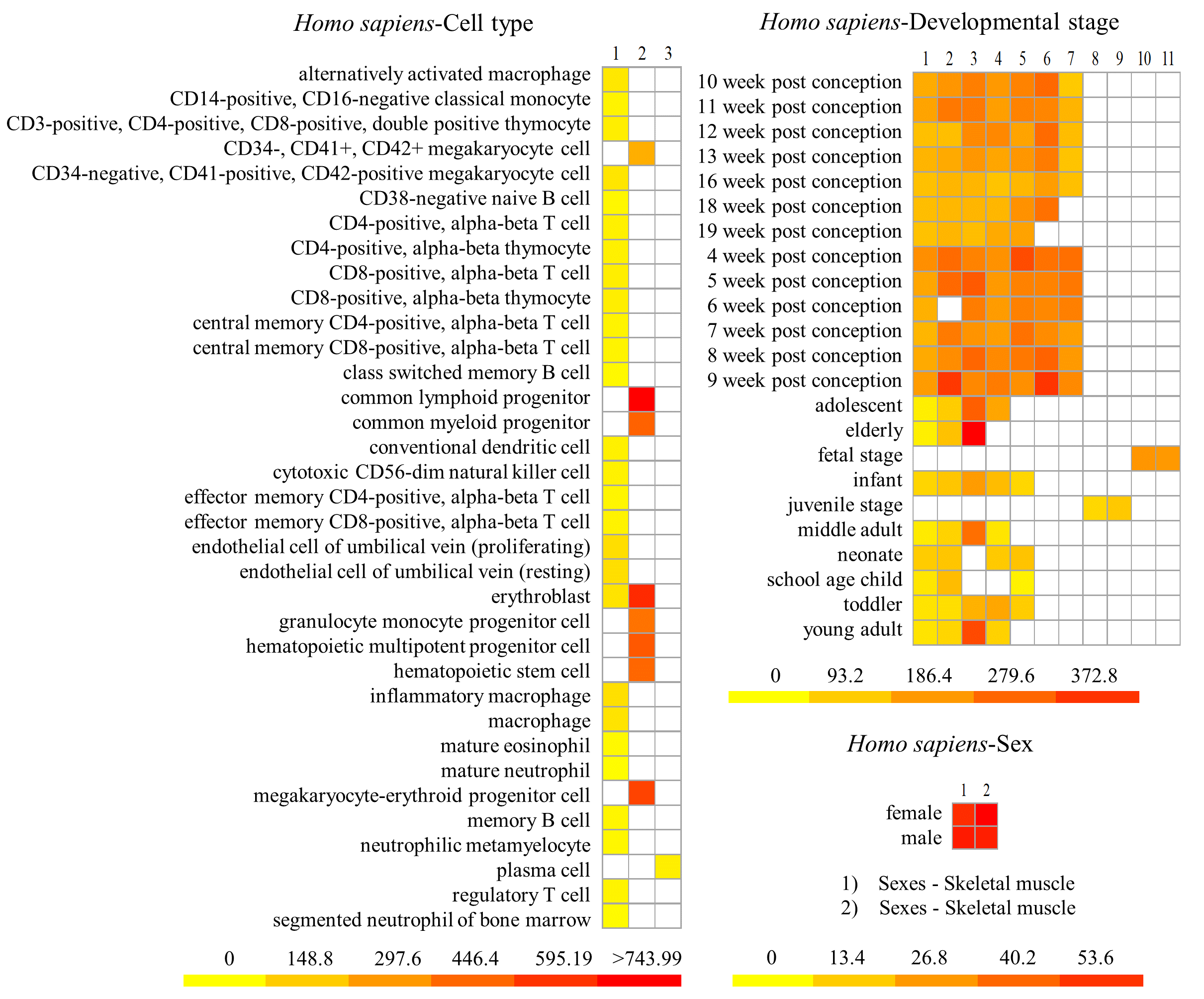


**Figure** **S11 Expression of human *U1C* in various** **cell types, developmental stages and** **sex.** The columns in the heatmaps show the available experiments in Expression Atlas. The rows in the heatmaps show experimental condition such as developmental stage, different tissue and cell type and disease condition. White box presents there is no data available. No.1-3 of *Homo sapiens*-cell type represent (1) Cell Types - BLUEPRINT common haemopoetic cells, (2) Cell Types - BLUEPRINT rare hematopoietic cells, (3) Cell Types - BLUEPRINT tonsil plasma cells. No.1-11 of *Homo sapiens*-developmental stage represent (1) Tissues, developmental stages - Human - Moreira et al - liver, (2) Tissues, developmental stages - Human - Moreira et al - forebrain Tissues, (3) developmental stages - Human - Moreira et al - testis, (4) Tissues, developmental stages - Human - Moreira et al - heart, (5) Tissues, developmental stages - Human - Moreira et al - kidney, (6) Tissues, developmental stages - Human - Moreira et al - ovary, (7) Tissues, developmental stages - Human - Moreira et al - hindbrain, (8) Kraiczy et al intestinal cells - terminal ileum, (9) Kraiczy et al intestinal cells - sigmoid colon, (10) Kraiczy et al intestinal cells - proximal gut, (11) Kraiczy et al intestinal cells - distal gut, respectively. Data are all from RNA-seq experiments. Baseline expression levels from RNA-seq experiments are in TPM (transcripts per million). References for each project refer to Table S6.


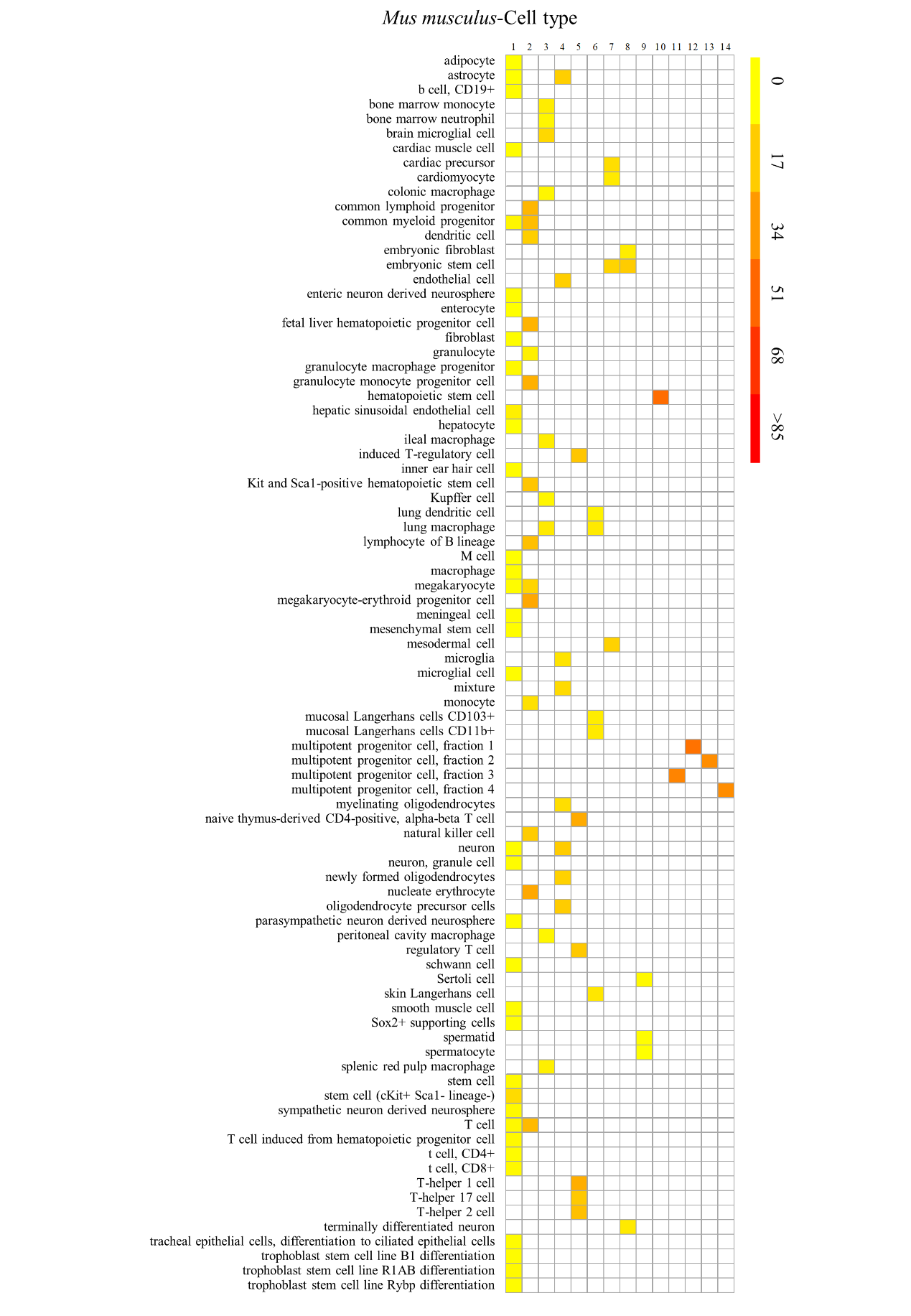
 **Figure S12 Expression of mouse *U1C* in cell type.** The columns in the heatmaps show the available experiments in Expression Atlas. The rows in the heatmaps show experimental condition such as developmental stage, different tissue and cell type and disease condition. White box presents there is no data available. No.1-14 represent (1) Cell Types - 35 FANTOM5 project, (2) Cell type - Paulson et al, (3) Cell types - Lavin et al, (4) Cell Types - Cerebral cortex, (5) Cell Types - Six T cell subtypes, (6) Cell types - Capucha et al, (7) Cell type - Wamstad et al, (8) Cell types - 3 Lienert et al, (9) Cell type - Soumillon et al, (10) Cell type - Cabezas-Wallscheid et al - Lin neg Sca-1+ c-Kit+, LSK, CD34- CD135- CD150+ CD48-, (11) Cell type - Cabezas-Wallscheid et al - LSK CD34+ CD135- CD150- CD48+, (12) Cell type - Cabezas-Wallscheid et al - LSK CD34+ CD135- CD150+ CD48-, (13) Cell type - Cabezas-Wallscheid et al - LSK CD34+ CD135- CD150+ CD48+, (14) Cell type - Cabezas-Wallscheid et al - LSK CD34+ CD135+ CD150- CD48+, , respectively. Baseline expression levels from RNA-seq experiments are in TPM (transcripts per million). References for each project refer to Table S6.


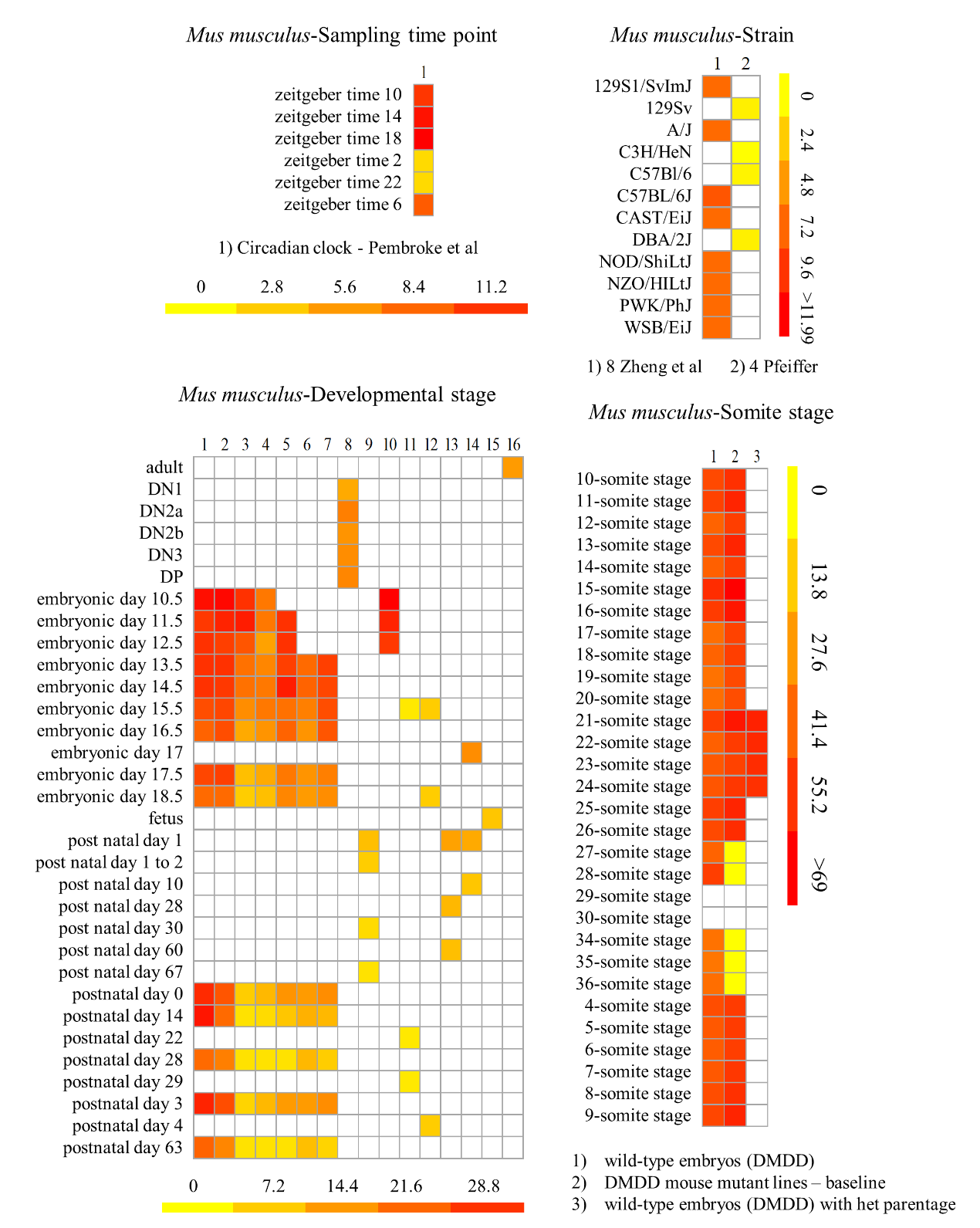


**Figure** **S13 Expression of** **mouse *U1C* in various** **sampling time points,** **strains,** **developmental stage and** **somite stages.** The columns in the heatmaps show the available experiments in Expression Atlas. The rows in the heatmaps show experimental condition such as developmental stage, different tissue and cell type and disease condition. White box presents there is no data available. No.1-16 of *Mus musculus*- developmental stage represent (1) Tissues, developmental stages - Mouse - Moreira et al - testis, (2) Tissues, developmental stages - Mouse - Moreira et al - ovary, (3) Tissues, developmental stages - Mouse - Moreira et al - liver, (4) Tissues, developmental stages - Mouse - Moreira et al - heart, (5) Tissues, developmental stages - Mouse - Moreira et al - kidney, (6) Tissues, developmental stages - Mouse - Moreira et al - hindbrain, (7) Tissues, developmental stages - Mouse - Moreira et al - forebrain, (8) 5, (9) Cell Types - Developing heart - cardiomyocyte, (10) Tissues, developmental stages - Mouse - Moreira et al - brain, (11) Developmental stages - Schmitt et al - liver, (12) Developmental stages - Schmitt et al - brain, (13) Cell Types - Developing heart - fibroblast, (14) Cell Types - Developing heart - cells of ventricle, (15) Developmental stages - 4 - liver, (16) Developmental stages - 4 - bone marrow, respectively. Baseline expression levels from RNA-seq experiments are in TPM (transcripts per million). References for each project refer to Table S6.


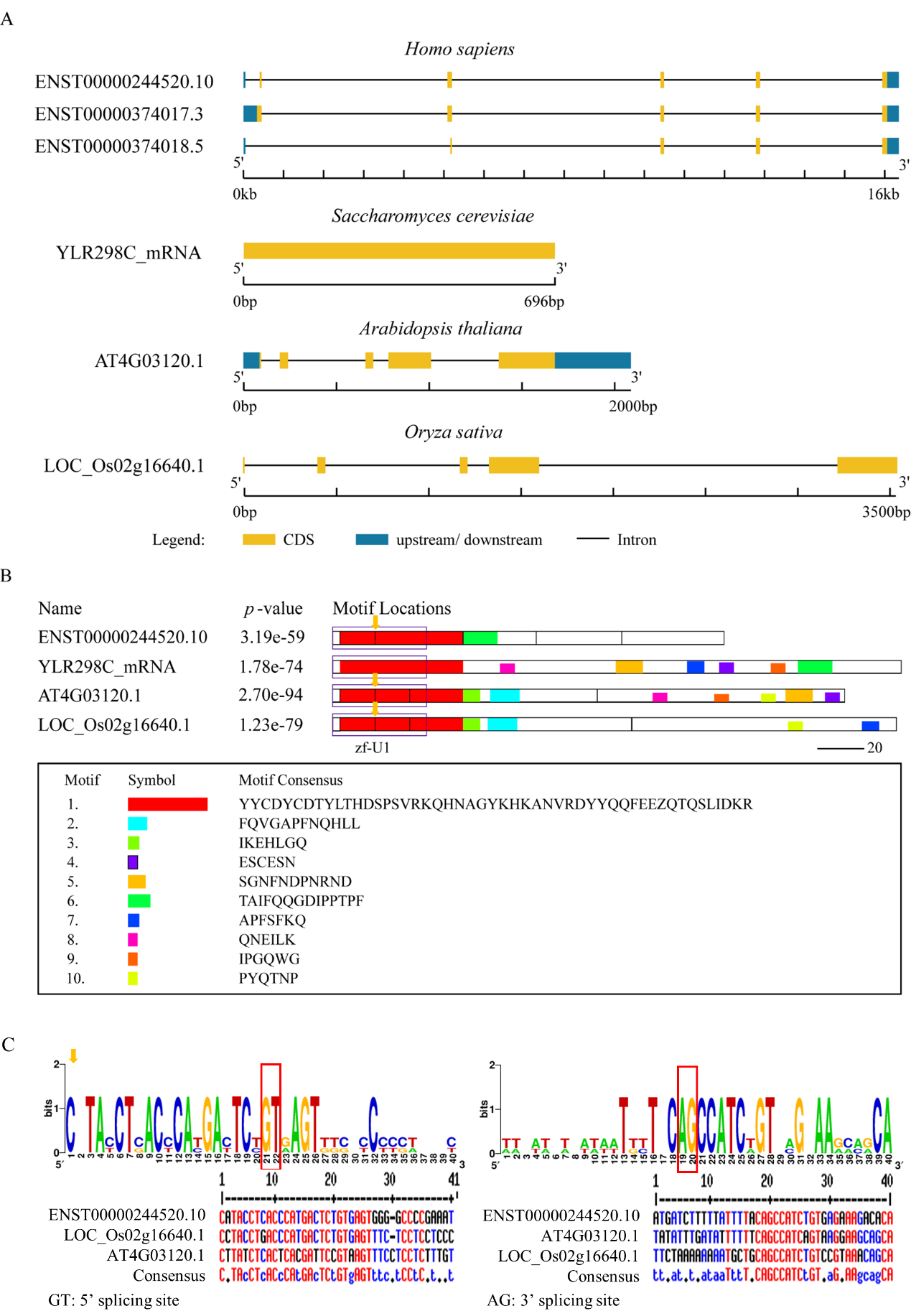


**Figure S14 Comparison of U1Cs in human, yeast, Arabidopsis and rice. (A)** Representation of gene structures of *U1Cs* in human, yeast, Arabidopsis and rice. **(B)** Conserved motif analysis of human and Arabidopsis U1Cs. Black boxes indicate exon-intron boundaries. **(C)** Conserved splice sites analysis of human, Arabidopsis and rice U1Cs. Orange arrow represents conserved splice sites. Red boxes represent 5’ splice sites and 3’ splice sites.
